# Additive Effects of Retinoic Acid (RA) and Bone Morphogenetic Protein 4 (BMP-4) Apoptosis Signaling in Retinoblastoma Cell Lines

**DOI:** 10.1371/journal.pone.0131467

**Published:** 2015-07-14

**Authors:** Patrick Müller, Rebekka Doliva, Maike Busch, Claudia Philippeit, Harald Stephan, Nicole Dünker

**Affiliations:** 1 Department of Neuroanatomy, Institute for Anatomy, Medical Faculty, University of Duisburg-Essen, Essen, Germany; 2 Division of Haematology and Oncology, Children’s Hospital, University of Duisburg-Essen, Essen, Germany; Nihon University School of Medicine, JAPAN

## Abstract

Retinoids have been shown to serve promising therapeutic agents for human cancers, e.g. the treatment of neuroblastoma. Synthetic retinoids, specific for particular retinoic acid (RA) receptors, are tested as new therapy strategies. In the present study, application of recombinant retinoic acid (RA) lowers retinoblastoma (RB) cell viability and induces apoptosis in RB cell lines. Combined treatment of RA and bone morphogenetic protein 4 (BMP-4) increases the pro-apoptotic effect of RA in the RB cells lines WERI-Rb1, Y-79, RB355, RBL-30 and RBL-15, indicating an additive effect. We could show that in WERI-Rb1 cells RA/BMP-4 mediated cell death is at least partially caspase-dependent, whereby RA and BMP-4 additively increased (i) Apaf-1 mRNA levels, (ii) caspase-9 cleavage activity and (iii) the number of activated, cleaved caspase-3 positive cells. Compared to single application of RA and BMP-4, combined RA/BMP-4 treatment significantly augments mRNA levels of the retinoic acid receptors (RARs) *RARα* and *RARß* and the retinoic X receptor (RXR) *RXRγ* suggesting an interaction in the induction of these RA receptor subtypes in WERI-Rb1 cells. Agonist studies revealed that both, RARs and RXRs are involved in RA/BMP-4 mediated apoptosis in WERI-Rb1 retinoblastoma cells. Employing specific RAR subtype antagonists and a *RXRß* and *RXRγ* knockdown, we proved that RA/BMP-4 apoptosis signaling in WERI-Rb1 cells requires the RA receptor subtypes RARα, RARß, RXRß and RXRγ. Deciphering signaling mechanisms underlying apoptosis induction of RA and BMP-4 in WERI-Rb1 cells, our study provides useful starting-points for future retinoid-based therapy strategies in retinoblastoma.

## Introduction

Retinoids, natural and synthetic vitamin A derivatives, are known to inhibit tumor growth and to suppress carcinogenesis, e.g. in MCF-7 breast cancer and Hep 3B cells [1; 2].

The effects of retinoids are mediated by two classes of nuclear receptors, the retinoic acid receptors (RARs) and the retinoic X receptors (RXRs). RARs are ligand-controlled transcription factors forming heterodimers with RXRs that regulate cell growth, differentiation, survival and death [3; 4]. RARs and RXRs modulate the expression of their target genes by binding to specific retinoic acid response elements (RAREs) [5; 6]. All-*trans*-RA acid (ATRA) directly activates only RARs. However, in some cells ATRA is converted to 9-*cis*-RA and this retinoid directly activates RARs and RXRs [7; 8]. Due to their regulatory potential, RARs and RXRs are major drug targets for a variety of pathologies, including cancer. Each subtype of RARs has been implicated in the regulation of cancer development and the anticancer activities of retinoids [9]. *RARß* is a tumor suppressor gene [10] and the best characterized RA responsive receptor with a confirmed ßRARE binding site. Former studies indicated that up-regulation of the *RARß* gene plays a critical role in mediating the apoptosis-inducing effect of retinoids in many different types of cancer cells [11–13]. A large amount of RAR- and RXR-selective ligands, ranging from agonists to antagonists have been designed [14] and are tested as new retinoid-based therapy strategies [3; 15]. Thus, retinoids serve as promising therapeutic agents for many human cancers [9; 16–19].

BMPs are members of the transforming growth factor beta (TGF-ß) family, originally identified by their bone-inducing activities. We and others could, however, show that BMPs are also involved in other scenarios besides osteogenesis, e.g. the induction of apoptosis [20]. Former studies demonstrated that BMP-4 and RA synergistically induce apoptosis in P19 embryonal carcinoma cells [21; 22]. If this also holds true for retinoblastoma cells and which molecular mechanisms play a role in a potential synergistic or additive apoptosis induction in RB cells has not been investigated so far.

Against the background to develop novel mechanism-based approaches using retinoids in the prospective treatment of retinoblastoma, in the present study we set out to determine the effects of exogenous RA and combined RA/BMP-4 application on WERI-Rb1 retinoblastoma cell viability and apoptosis and to elucidate signaling mechanism underlying these effects, including the involvement of RARs and RXRs, specific RA receptor subtypes and caspases. Deciphering signaling mechanisms underlying apoptosis induction of RA and BMP-4 in WERI-Rb1 cells, our study provides useful starting-points for future retinoid-based therapy strategies in retinoblastoma.

## Materials and Methods

### Cell culture

The Rb cell lines RB355 and RB383 (originally established by B. Gallie) and the cell lines RBL-13, RBL-15 and RBL-30, established and first described by Griegel et al. [23] and formerly donated by K. Heise, were kindly provided by Dr. H. Stephan. The human retinoblastoma cell lines Y-79 [24] and WERI-Rb1 [25] originally purchased from the Leibniz Institute DSMZ (German Collection of Microorganisms and Cell Cultures) were kindly provided by Dr. H. Stephan. The cell lines were cultivated as suspension cultures in Dulbecco’s modified Eagle’s medium (DMEM; PAN-Biotech) with 10% fetal calf serum (FCS; PAN-Biotech), 100 U penicillin/ml and 100 μg streptomycin/ml (Invitrogen), 4 mM L-glutamine (Sigma), 50 μM ß-mercaptoethanol (Roth) and 10 μg insulin/ml (Sigma) at 37°C, 10% CO_2_ and 95% humidity. Cells were treated with (i) 1–40 ng/ml of recombinant human BMP-4 (R&D Systems), (ii) 0.005–10 μM of all-*trans* retinoic acid (ATRA; Sigma; stock: 10 mM in 99.8% ethanol) or (iii) a combination of both for 24 h–48 h. Cells treated with equivalent amounts of HCl/BSA and ethanol vehicle, respectively, were used as controls. For immunocytochemistry, cells were seeded on poly-D-lysine coated (Sigma) coverslips and fixed with 4% paraformaldehyde for 1 h at 4°C.

### Cell proliferation and apoptosis detection

In order to determine effects on cell proliferation and apoptosis by 5-bromo-2'-deoxyuridine (BrdU) incorporation, 4',6-diamidino-2-phenylindole (DAPI)-staining and Apo-BrdU TUNEL assay (Life Technologies), 1 x 10^5^ WERI-Rb1 cells, synchronized by serum-starvation, were seeded on cover slips and cultivated in 24-well plates in 300 μl medium containing 5% FCS in the presence of single additives or treated with a combination of several substances as summarized in Table 1. Medium without insulin was used as insulin has been shown to interfere with apoptosis induction (personal observation).

**Table 1:** Application scheme.

	retinoic acid (RA)	BMP-4	inhibitor / agonists / antagonists
**1**	5 μM RA	-	**-**
**2**	**-**	40 ng/ml BMP-4	**-**
**3**	5 μM RA	40 ng/ml BMP-4	**-**
**4**	5 μM RA	-	38 μM Boc-D-fmk^a^
**6**	5 μM RA	40 ng/ml BMP-4	38 μM Boc-D-fmk
**7**	-	-	5 μM TTNPB^b^
**8**	**-**	40 ng/ml BMP-4	5 μM TTNPB
**10**	-	-	5 μM methoprene acid^c^
**11**	**-**	40 ng/ml BMP-4	5 μM methoprene acid
**13**	5 μM RA	-	10 μM ER50891^d^
**15**	5 μM RA	40 ng/ml BMP-4	10 μM ER50891
**16**	5 μM RA	-	10 μM LE135^e^
**18**	5 μM RA	40 ng/ml BMP-4	10 μM LE135

^a^BOC-D-fmk: caspase inhibitor

^b^TTNPB: RAR agonist

^c^methoprene acid: RXR agonist

^d^ER50891: RARα antagonist

^e^LE135: RARß antagonist.

For the determination of effects on cell proliferation, BrdU (5 μM; Sigma) was added 6 h before the end of culture time and BrdU immunocytochemistry was performed as described previously [20]. BrdU positive cells and 4',6-diamidino-2-phenylindole (DAPI)-stained pycnotic nuclei were counted microscopically. For this purpose, at least 10 different fields of view of one coverslip and at least 1000 cells were counted and the number of DAPI-positive, clearly pycnotic cells with condensed nuclei or obvious apoptotic bodies (at least 10) or clearly BrdU-positive stained cells was determined.

### WST-1 assay

The WST-1 assay is based on the cleavage of the tetrazolium salt WST-1 to a water soluble formazan by cellular mitochondrial dehydrogenases. Expansion in the number of viable cells results in an increase in the overall activity of the mitochondrial dehydrogenases in the sample. For the determination of cell viability, 4 x 10^4^ serum starved WERI-Rb1 cells were seeded in 96-well plates in 100 μl medium without insulin containing 5% FCS to release cells from growth arrest and various concentrations (0.005, 0.05, 0.5, 5 and 10 μM) RA or respective concentrations of ethanol (controls) were applied. 48 h later, WST-1 reagent was added and the water-soluble formazan dye produced by viable cells was quantified by measuring the absorbance at 440 nm in a microplate reader after 120 min.

### Flow cytometry

For cell cycle analysis, cells were suspended in 10 mM Tris-hydrochloride (pH 7.5) / 5 mM MgCl_2_ / 100 μg/ml propidium iodide and analyzed in a Cytomics FC500 flow cytometer using CXP software (Beckman-Coulter, Germany). The percentage of cells present in the sub-G0/G1 peak, representing apoptotic cells, was calculated after exclusion of cell doublets.

### Inhibition of endogenous caspase activity

In order to block endogenous caspase activity, Boc-D-fmk (Merck, Germany), a broad spectrum caspase inhibitor was used. WERI-Rb1 cells were seeded on Poly-D-lysine coated coverslips and pre-incubated with 38 μM Boc-D-fmk or DMSO as solvent control for 30 min. Afterwards cells were incubated for 24 h in DMEM without insulin containing 5% FCS supplemented with 5 μM RA and 5 μM RA + 40 ng/ml BMP-4. The number of pycnotic nuclei was assessed by DAPI staining (see above).

### Assay for caspase-9 cleavage activity

WERI-Rb1 cells were treated for 8 h with 5 μM RA, 40 ng/ml BMP-4 and a combination of both in 300 μl 5% FCS containing medium. The cleavage activity of caspase 9 was analyzed using the Caspase-Glo 9 Assay (Promega) according to the manufacturer’s protocol. Each measurement was performed in triplicate. As a positive control, cells treated for 3 h with 50 μM staurosporine were used.

### Immunocytochemistry

For immunostaining of active caspase-3 1 x 10^5^ serum starved WERI-Rb1 cells were seeded on cover slips and cultivated in 24-well plates in 300 μl 5% FCS containing medium without insulin in the presence of (i) 5 μM RA, (ii) 40 ng/ml recombinant BMP-4, or (iii) 5 μM RA + 40 ng/ml BMP-4. Cells were fixed with 4% PFA for 1 h, followed by three washes with PBS. Afterwards, cells were fixed with ice-cold 100% methanol for 5 min on ice, washed three times with PBS and incubated for 1 h in blocking solution (0.3% Triton X-100, 4% BSA, 5% normal goat serum (NGS; Dako in PBS). A rabbit monoclonal cleaved caspase-3 antibody (5A1E; Cell Signaling, Danvers MA) was used as a primary antibody and diluted 1:400 in PBS containing 0.1% Triton X100, 4% BSA, and 1% NGS at 4°C overnight. Following three washes with PBS, a species-specific Alexa-594-coupled secondary antibody (Molecular Probes, Life Technologies) was used diluted 1:1,000 in PBS with 1% BSA. Cover slips were embedded in fluorescence mounting medium (Dako) containing DAPI. As controls, in all cases PBS was substituted for the primary antisera in order to test for nonspecific labeling. No specific cellular staining was observed when the primary antiserum was omitted.

### Agonist and antagonist studies

The following RAR and RXR agonists and antagonists were used: general RAR agonist TTNPB (4-[(E)-2-(5,6,7,8-Tetrahydro-5,5,8,8-tetramethyl-2-naphthalenyl)-1-propenyl] benzoic acid; Sigma-Aldrich) [26]; general RXR agonist methoprene acid (2E,4E-11-Methoxy-3,7,11-trimethyl-2E,4E-dodecadienoic acid; Enzo Life Sciences) [27; 28]; specific RARα antagonist (ER 50891; Tocris Bioscience) [29] and specific RARß antagonist (LE 135; Tocris Bioscience) [30].

Retinoids were prepared as 10 mM (agonists) or 2.5 mM (antagonists) stock solutions in DMSO. WERI-Rb1 cells were treated for 48 h with 5 μM TTNPB or methoprene acid and with 10 μM of the RAR antagonists LE135 and ER50891, then fixed with 4% paraformaldehyde for 1h at 4°C and stained with DAPI. Control cells were treated for 48 h with an equivalent amount of DMSO, the solvent used for the agonists and antagonists.

### Lentiviral expression vectors, production of lentiviral particles and tranduction

For the RXRß and RXRγ knockdown experiments a “Mission shRNA Plasmid DNA” with a pLKO.1puro backbone was used (Sigma-Aldrich; shRXRß: clone# TRCN0000021628; shRXRγ: clone# TRCN0000021639). A pPRIME-CMV-Neo-FF3 (p234) vector containing a targeting hairpin sequence against firefly luciferase served as a non-mammalian sh control [31].

For virus production HEK293T cells were transfected with 6 μg of each plasmid DNA: (a) packaging vectors pczVSV-G, pCD NL-BH [32] and (b) the desired expression vector in the presence of 45 μg PEI (Polyethyleneimine, branched, Aldrich). The medium was changed after one day to IMDM with 10% FCS and 1% penicillin/streptomycin and next day viral supernatants were harvested, sterile-filtered (0.45 μM) and cryoconserved.

For the RXRß and RXRγ knockdown experiments WERI-Rb1 cells were seeded in a 6 well plate (3 x 10^5^ cells/well in 2 ml growth medium as described above). After 24 h the cells were infected with 1 ml shRXRß or shRXRγ lentiviruses, respectively or with 1 ml lentiviruses expressing a non-silencing shRNA in the presence of polybrene (5 μg/ml, H9268; Sigma), serving as a control. After 24 h, additional 2 ml of normal growth medium was added to the cells, and after another 48 h the medium was completely changed. RNA was isolated and DAPI stains were performed six days after transduction. The experiments were performed in duplicates.

### RNA isolation and Quantitative Real-time PCR

RNA isolations from the RB cell lines were performed using the NucleoSpin RNA II kit (Machery and Nagel), and cDNA was synthesized with the QuantiTect Reverse Transcription Kit (Qiagen) following the manufacturer’s protocols. Pooled human adult total retinal RNA, used as a reference, was purchased from Clontech (cat.# 636579). Quantitative Real-time PCR analyses were performed using a 7300 Real-Time PCR System (Applied Biosystems). The following Taqman Gene Expression Assays (Applied Biosystems) were used: RARα: Hs00940446_m1; RARß: Hs00977140_m1; RARγ: Hs00199455_m1; RXRγ: Hs00199455_m1; and Apaf-1: Hs00559421_m1. GAPDH (ID Hs99999905_m1) as an endogenous control. Real-time PCR reactions were performed in duplicate with a total volume of 20 μl applied to the following program: 2 min 50°C, 10 min 95°C, followed by 40 cycles of 15 sec 95°C and 60 sec 60°C. Relative quantification was calculated by the 7300 Real-Time PCR System software (Applied Biosystems). Results represent mean values of at least four independent experiments.

### Statistical analysis

All assays were performed in triplicate. Data represent means ± SEM of at least three independent RNA preparations from independent RB cell cultures. Results were analysed by a Student`s *t*-test or one way Annova and Newman-Keuls Post test. Values were considered significantly different if *P<0.05, **P<0.01 or ***P<0.001.

## Results

### RA lowers cell viability and induces apoptosis in WERI-Rb1 cells

**Fig 1:**
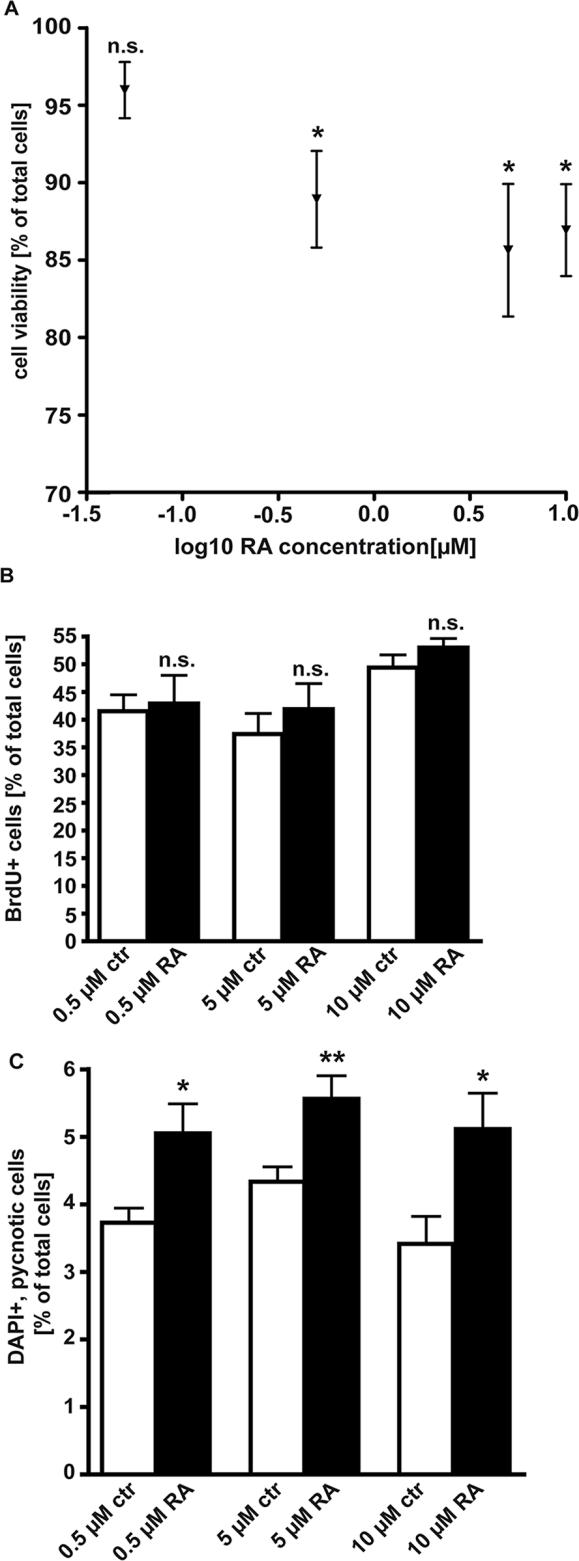
Effect of RA treatment on cell viability, cell proliferation and cell death in the retinoblastoma cell line WERI-Rb1. Cells were grown in medium containing indicated concentrations of RA or ethanol (control; set as 100%). Application of RA decreased the viability of WERI-Rb1 cells as determined by WST-1 assays (A). Cell counts from BrdU incorporation experiments (B) and counts of DAPI-positive, pycnotic nuclei (C) were performed to determine proliferation and apoptosis rates, respectively. Application of increasing amounts of RA had no significant effect on the number of BrdU-positive, proliferating cells (B), but significantly induced apoptosis in WERI-Rb1 cells (C).*P < 0.05; **P < 0.01 significant statistical differences compared to the control group calculated by Student`s *t*-test. n.s.: no significant statistical difference.

We set out to determine the effect of increasing concentrations of exogenously applied RA on RB cell viability. In WST-1 assays, 0.5 μM and 5 μM RA significantly lowered WERI-Rb1 cell viability to values between 84% and 87%, whereby higher concentrations of RA (10μM) were not able to further increase this effect (Fig 1A). In order to determine if this significant decrease in cell viability is due to a decrease in cell proliferation or rather an increase in apoptosis, we counted the number of BrdU-stained cells (Fig 1B) and DAPI-positive, apoptotic nuclei (Fig 1C), respectively. Application of increasing amounts of RA had no significant effect on the number of BrdU-positive, proliferating cells (Fig 1B), but significantly induced apoptosis in WERI-Rb1 cells (Fig 1C). As the application of 0.05 μM RA did not exert a significant effect in our WST-1 assays, this concentration was not included in the BrdU and DAPI cell count approaches.

### RA and BMP-4 additively induce apoptosis in retinoblastoma cell lines

In the present study, the effect of RA on cell survival and apoptosis in WERI Rb1 cells was only moderate and the effect of BMP-4, determined in a former study by our group [20], though significant, was likewise low. In this context, other groups showed that a combination of BMP-4 and RA significantly increased apoptosis in cells, in which application of RA and BMP-4 alone only exerted small apoptotic effects [21; 22] and that this synergistic apoptosis induction is dose-dependent.

**Fig 2:**
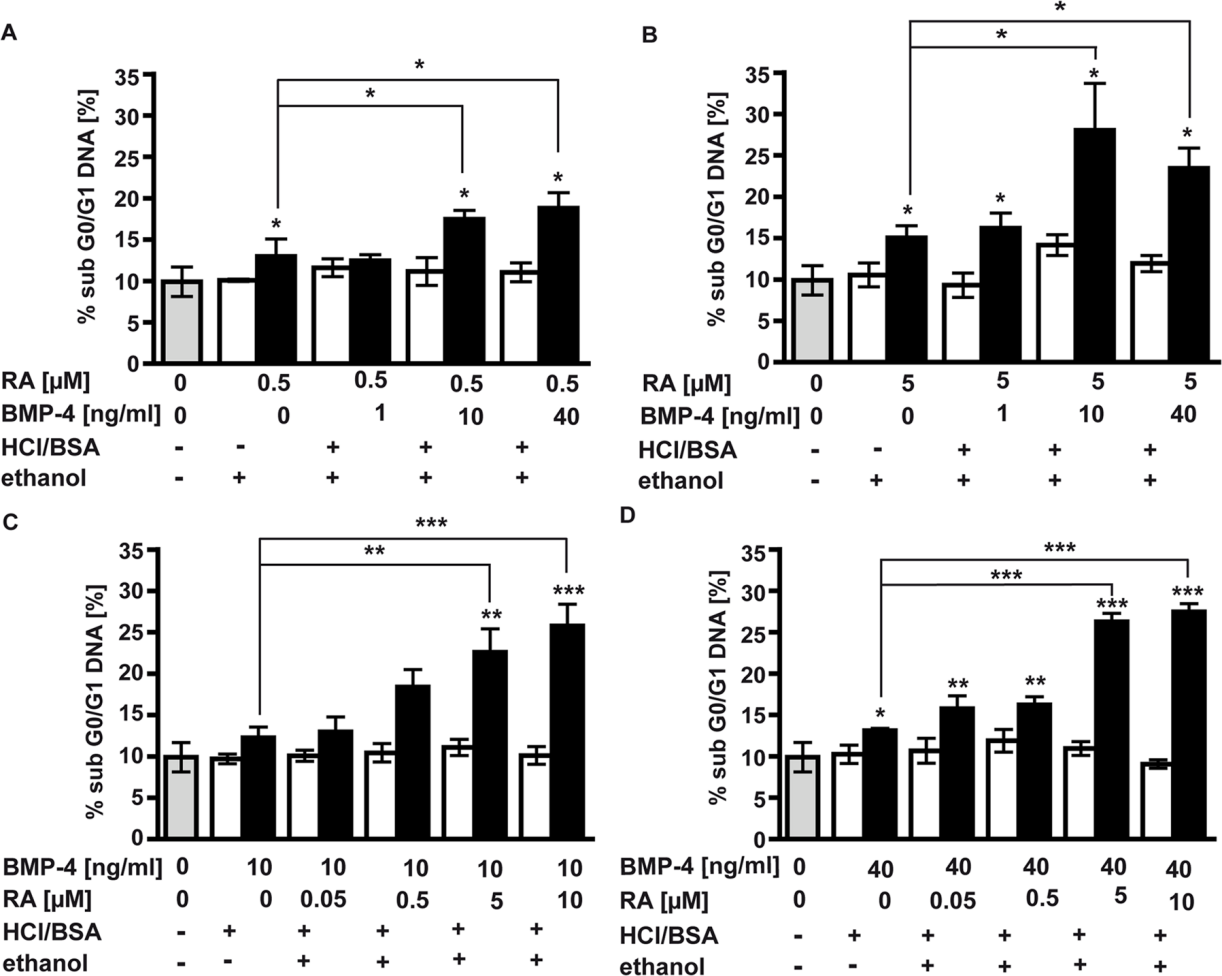
FACS analyses of WERI-Rb1 cells. WERI-Rb1 cells were treated for 48 h with two fix concentrations of RA (0.5μM and 5μM; A,B) or BMP-4 (10 ng/ ml and 40 ng/ml; C,D) and rising concentrations of BMP-4 (1–40 ng/ml) or RA (0.05–10 μM), respectively. Under these conditions, RA and BMP-4 additively and dose-dependently induced an increase in subG0/G1, representing apoptotic cells. Gray bars: untreated control cells; white bars: cells treated (+) or not (-) with equivalent concentrations of HCl/BSA, the solvent for BMP-4, ethanol, the solvent for RA, or both. Values are means from 3–4 independent assays ± SEM. *P < 0.05, **P < 0.01, ***P < 0.001 statistical differences calculated by one way Annova and Newman-Keuls Post test.

We thus set out to investigate a possible synergistic or additive effect of combined RA and BMP-4 application in the induction of apoptosis in WERI-Rb1 cells and to reveal a potential concentration dependence of this effect. For this purpose, we added increasing amounts of BMP-4 to WERI-Rb1 cells treated with 0.5 μM (Fig 2A) or 5 μM RA (Fig 2B). While the addition of low concentrations of BMP-4 (1 ng/ml) resulted in no increase in the amount of subG0/G1 DNA, the addition of 10 ng/ml BMP-4 resulted in a significant increase in the amount of dead cells compared to RA single treatment (Fig 2A and 2B).

In a second set of experiments, we titrated the RA concentration in the presence of 10 ng/ml and 40 ng/ml BMP-4. As shown in Fig 2C and 2D, there is also a dose-dependent response to RA in that the addition of higher concentrations of RA (5 μM and 10 μM) resulted in a higher proportion of fragmented DNA, whereas low concentrations of RA (0.05 μM and 0.5 μM) did not increase the number of cells in the sub G0/G1 fraction. Thus, combined application of RA and BMP-4 results in a dose-dependent increase of the pro-apoptotic effect obtained by single factor treatment, indicating an additive interaction of both factors in apoptosis induction in WERI-Rb1 cells.

BrdU cell counts indicated that RA/BMP-4 double treatment had no significant impact on WERI-Rb1 cell proliferation (S1 Fig).

In long-time experiments (S2 Fig), we detected slightly higher apoptosis levels 48 h after application of RA, BMP-4 or a combination of both, but only in double treatment approaches and only after re-stimulation (Figs. A and B in S2 Fig). Seventy-two hour treatment resulted in an increase in the number of apoptotic cells in single treatment approaches, whereas re-stimulation after 24 h and 48 h slightly augmented the pro-apoptotic effect of combined factor treatment (Figs. C and D in S2 Fig).

We performed additional Apo-BrdU TUNEL assays in order to validate our DAPI cell count data. Exemplary counts of TUNEL-positive WERI-Rb1 cells seeded on coverslips and counterstained with Propidium iodide, revealed apoptosis rates mirroring and thereby confirming our data from DAPI cell counts after RA, BMP-4 and combined treatment (S3 Fig).

DAPI cell counts in the four additional RB cell lines Y-79, RB355, RBL-30 and RBL-15 confirmed the data gained in WERI-Rb1 cells, generalizing the notion of a pro-apoptotic effect of RA, BMP-4 and combined treatment in RB cell lines (S4 Fig). Interestingly, in RBL-15 cells (Fig D in S4 Fig), derived from a bilateral tumor, apoptosis induction by combined RA/BMP-4 treatment was considerably higher compared to the effects observed in Y-79 (Fig A in S4 Fig), RB355 (Fig B in S4 Fig) and RBL-30 (Fig C in S4 Fig), established from unilateral retinoblastomas, more or less mirroring the pro-apoptotic effects obtained in WERI-Rb1 cells.

### Caspases are involved in RA and RA/BMP-4 induced apoptosis in WERI-Rb1

**Fig 3:**
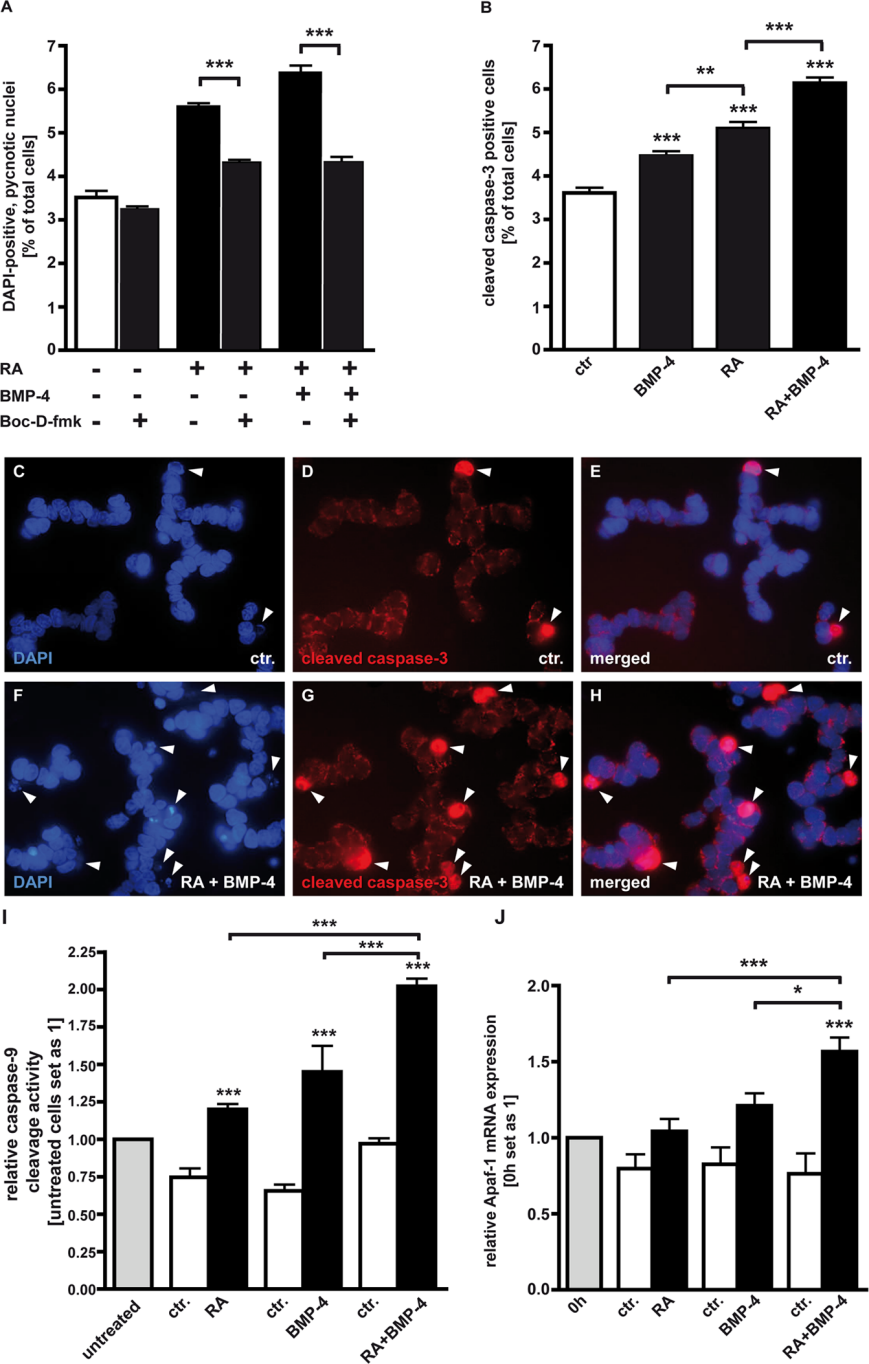
Involvement of caspases in RA and BMP-4 mediated apoptosis induction in WERI-Rb1 cells. A, Blockage experiments with a broad spectrum caspase inhibitor (Boc-D-fmk) in the presence (+) or absence (-) of RA and BMP-4. B, Quantification of cleaved (= activated) caspase-3 positive cells after treatment with RA, BMP-4 or both factors. C-H, Immunocytochemical caspase-3 staining. I, Up-regulation of caspase-9 cleavage activity following administration of RA, BMP-4 or a combination of both. Untreated cells, revealing the endogenous caspase-9 cleavage activity, served as a standard and were set as 1. Cells treated with the solvent for RA and BMP-4 (see material and method), respectively, served as controls (ctr.). J, Induction of *Apaf-1* upon application of RA, BMP-4 and RA+BMP-4 as revealed by Real-time PCR. Messenger RNA expression levels at the beginning of the treatment (0h) were used as a reference and set as 1. Values are means from 3 independent experiments ± SEM. *P < 0.05;**P < 0.01; ***P < 0.001 statistical differences calculated by Student`s *t*-test (A) or statistical differences calculated by one way Annova and Newman-Keuls Post test (B,I,J).

Previous studies by our group demonstrated that BMP-4 induced apoptosis in WERI-Rb1 cells is at least partially caspase-dependent [20]. In the present study, we further investigated the involvement of caspases in RA and RA/BMP-4 induced apoptosis in this retinoblastoma cell line using Boc-D-fmk, a broad spectrum caspase inhibitor. WERI-Rb1 cells were pre-incubated with Boc-D-fmk or DMSO, its solvent and pre-treated cells were incubated for 24 h in DMEM containing no insulin and only 5% FCS supplemented with 5 μM RA and 5 μM RA + 40 ng/ml BMP-4 (Fig 3A). By these experiments, we confirmed that in WERI-Rb1 cells endogenous apoptosis is caspase independent since the number of pycnotic nuclei was not significantly reduced in Boc-D-fmk treated cells compared to untreated controls (Fig 3A). DMSO, the solvent for Boc-D-fmk did not induce any significant changes in the number of DAPI-positive, pycnotic nuclei compared to untreated control cells in the concentration used (data not shown). Our data indicate that RA as well as RA/BMP-4 mediated cell death is at least partially caspase-dependent, as the number of pycnotic nuclei is significantly lower after BOC-D-fmk pre-treatment (Fig 3A).

As BMP-4 [20], RA, and RA/BMP-4 mediated apoptosis in WERI-Rb1 cells is at least partially caspase-dependent, we examined whether caspase-3 is activated by the respective treatments (Fig 3C and 3D). Immunocytochemical staining with a specific cleaved caspase-3 antibody (red immunofluorescence in Fig 3D and 3G) and counterstaining with DAPI (blue fluorescence in Fig 3C and 3F) revealed that WERI-Rb1 cells treated for 24 h with a combination of RA and BMP-4 displayed significant higher numbers of cleaved, activated caspase-3-positive cells (Fig 3H) compared to untreated control cells (Fig 3E). Quantification of cleaved caspase-3 positive cells (Fig 3B) proved that compared to RA and BMP-4 single treatment, RA/BMP-4 double treatment further increased the level of activated caspase-3 cells (Fig 3B), strongly suggesting that during apoptosis induction in WERI-Rb1 cells, RA and BMP-4 additively induce cleavage and thereby activation of caspase-3.

Cleavage activity of caspase-9—being upstream of caspase-3—significantly increased 8 h following administration of 5 μM RA or 40 ng/ml BMP-4 alone, and was further significantly increased by RA/BMP-4 double treatment (Fig 3I), strongly arguing for an additive enhancement of caspase-9 cleavage activity during RA/BMP-4 mediated apoptosis induction in WERI-Rb1 cells.

### *Apaf-1* is induced by RA/BMP-4 double treatment

Apaf-1 (apoptotic protease activating factor 1) molecules assemble into a key cell death platform known as the apoptosome that mediates the activation of pro-caspase-9, leading to activation of caspase-3 and apoptosis [33]. We thus set out to investigate if this key player in apoptosis signaling is regulated by RA, BMP-4 or additively by both factors. Our Real-time data revealed that *Apaf-1* mRNA levels remain unchanged upon treatment with 5 μM RA and 40 ng/ml BMP-4, whereas *Apaf-1* transcript levels significantly increased 48 h upon double treatment (Fig 3J), indicating an additive interaction also at this signaling level.

### RA and BMP-4 signaling up-regulates the expression of specific RAR and RXR subtypes

A ligand like RA can only mediate its effects when its receptors are expressed in the respective cell. Altered RA receptor levels determine the induction of apoptosis and are known to be associated with different cancers [34–36; 11]. Thus, in the study presented, we set out to determine if RB cell lines express all receptor subtypes essential for RA signaling. Analyzing *RARα*, *ß*, *γ* and *RXRα*, *ß*, *γ* subtype expression, we observed that all RB cell lines analyzed (RBL-13, RBL-15, RBL-30, RB355, RB383, WERI-Rb1, Y-79) constitutively express transcripts for all *RAR* and *RXR* receptor subtypes (S5 Fig).

**Fig 4:**
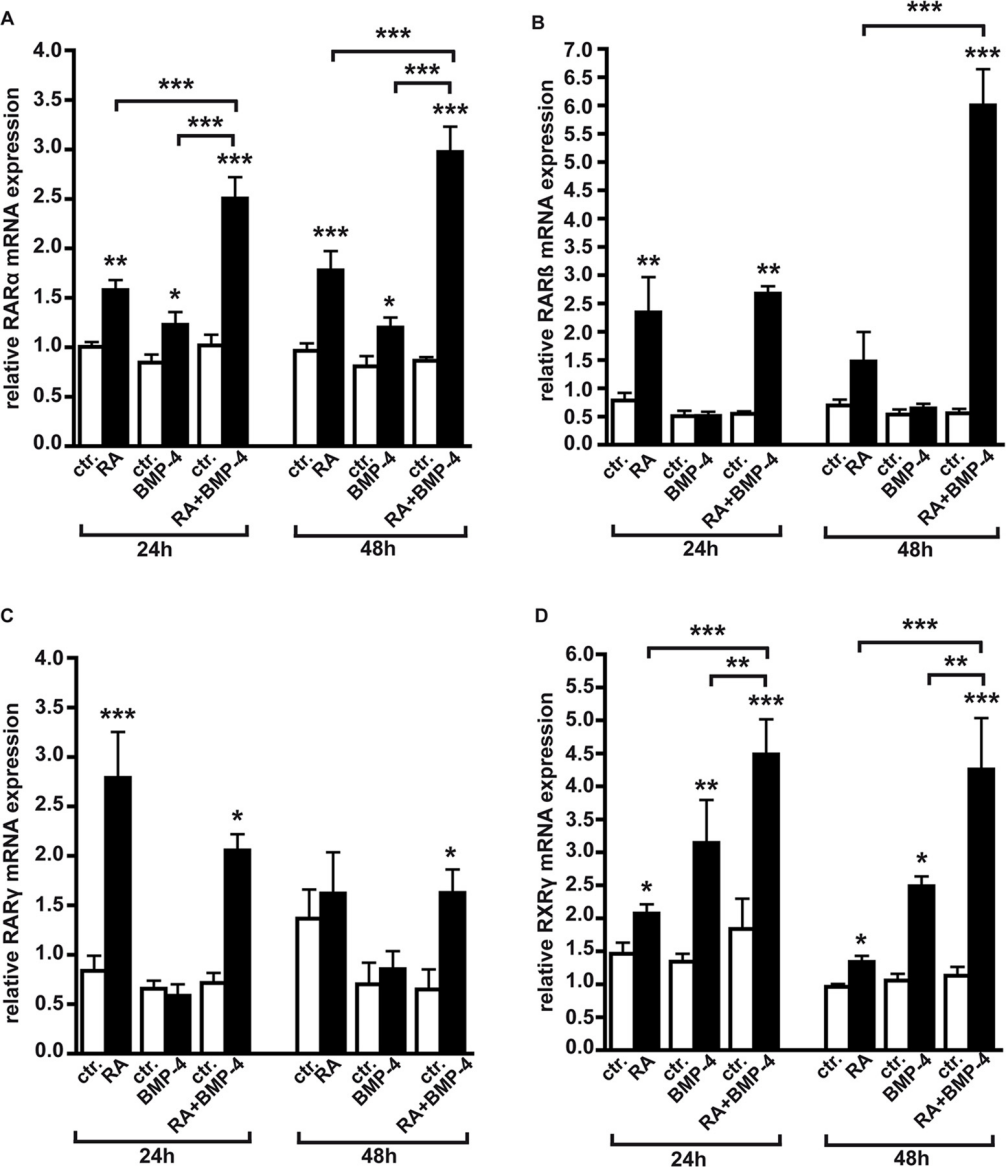
Induction of RAR and RXR subtypes by RA, BMP-4 and RA/BMP-4 double treatment in WERI-Rb1 cells as revealed by Real-time PCR. Messenger RNA expression levels at the beginning of the treatment (0h) were used as a reference and set as 1. The housekeeping gene GAPDH was used as an endogenous control. Data confirm an additive induction of *RARα* (A), *RARß* (B) and *RXRγ* (D) by RA/BMP-4 double treatment, whereas the up-regulation of *RARγ* (C) is solely attributable to RA. Values are means from four to five independent assays ± SEM. *P < 0.05; **P < 0.01; ***P < 0.001 statistical differences calculated by one way Annova and Newman-Keuls Post test.

Due to their regulatory potential, RARs and RXRs are major drug targets for cancer therapies. Thus, we next set out to investigate the effects of RA, BMP-4 and RA/BMP-4 double treatment on RAR and RXR subtype expression. As revealed by Real-time PCR, *RARα* levels significantly increased upon administration of RA (Fig 4A). Treatment of WERI-Rb1 cells with 40 ng/ml BMP-4, shown to be the optimal concentration to induce effects in this cell line [20], likewise significantly up-regulated *RARα* levels after 24 h and 48 h (Fig 4A), and RA/BMP-4 double treatment further augmented *RARα* mRNA expression, suggesting an additive interaction in which the factors boost each other’s effect.

*RARß* is significantly up-regulated 24 h after exogenous application of RA (Fig 4B). Co-application of BMP-4, which had no significant effect when applied alone, significantly increased this effect in RA/BMP-4 double treatment settings. Forty-eight hours after single application of RA or BMP-4 no significant effect on *RARß* expression was detectable, whereas *RARß* mRNA levels were more than doubled by RA/BMP-4 double treatment, strongly indicating an synergistic interaction of RA and BMP-4 in *RARß* induction.

*RARγ* is significantly up-regulated 24 h upon RA administration, but this modulatory effect could not be enhanced by additional application of BMP-4, which likewise had no influence on *RARγ* levels when applied alone (Fig 4C). Thus, the up-regulation seen upon RA/BMP-4 double treatment is solely attributable to RA.

It turned out that *RXRα* is not inducible neither by application of RA or BMP-4 alone, nor by RA/BMP-4 double treatment (Fig A in S6 Fig).

*RXRß* is significantly up-regulated by BMP-4, whereas its level remained unchanged upon application of RA (Fig B in S6 Fig). BMP-4`s effect could not be increased by additional application of RA in double treatment experiments (Fig B in S6 Fig), indicating that RXRß is solely regulated by BMP-4.

*RXRγ* mRNA levels significantly increased 24 h upon administration of RA and BMP-4 (Fig 4D). RA/BMP-4 double treatment further enhanced RA`s and BMP-4`s effect significantly, indicating that *RXRγ* expression is additively regulated by both agents.

We used the housekeeping gene GAPDH as an endogenous control in our RT- and Real-time PCR analyses. In order to verify that RA/BMP-4 treatment does not significantly modify the levels of GAPDH in WERI-Rb1 cells, we performed Real-time PCR analyses with additional housekeeping genes. Our data confirm that the additive induction of RA receptor mRNAs by RA/BMP-4 treatment—exemplified for the induction *RARα*, *RARß* and *RARγ* after 24 h – persists normalizing transcript levels against 18S rRNA or actin expression (S7 Fig).

### Apoptosis induction by RA and BMP-4 depends on the activation of RARs and RXRs

**Fig 5:**
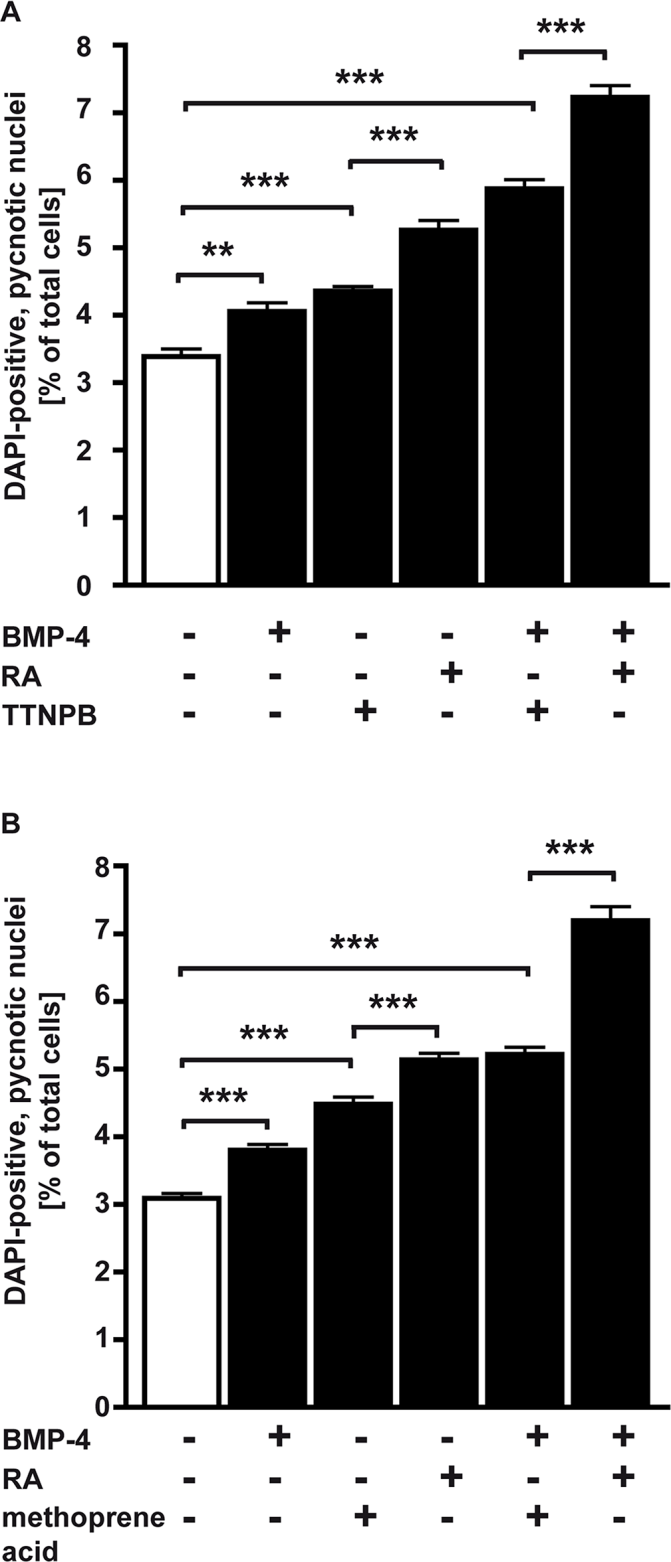
RAR and RXR agonist studies. Using 5 μM TTNPB, a stable RAR agonist (A) and 5 μM methoprene acid, a RXR agonist (B) in the presence (+) or absence (-) of RA and BMP-4 revealed the involvement of both RA receptor subtypes in RA and RA/BMP-4 mediated apoptosis induction in WERI-Rb1 cells. Values are means from two to three independent experiments ± SEM. **P < 0.01; ***P < 0.001 statistical differences calculated by one way Annova and Newman-Keuls Post test.

ATRA can directly activate only RARs; however, in some cells ATRA is converted to 9-*cis*-RA [37] and this retinoid can directly activate RARs and RXRs [7; 8]. Since apoptosis in e.g. HL-60 cells requires both, RAR and RXR activation [38], we set out to determine the role of each receptor class in RA and RA/BMP-4 mediated apoptosis induction of WERI-Rb1 cells. It is unknown whether metabolism of ATRA to 9-*cis*-RA might provide an RXR signal in WERI-Rb1 cells and we therefore used TTNPB, a stable RAR agonist and methoprene acid, a RXR agonist to assess the requirement for RAR and RXR activation during RA- and RA/BMP-4 induced apoptosis (Fig 5). TTNPB alone and in combination with BMP-4 clearly induced apoptosis 48 h after application, but the level of DAPI-positive, pycnotic nuclei were significantly lower compared to RA and RA/BMP-4 treated cells, indicating that RA and RA/BMP-4 induced apoptosis in WERI-Rb1 cells is mediated by RARs, but concomitant activation of RXRs is required to trigger the full pro-apoptotic effect (Fig 5A). Methoprene acid likewise induced apoptosis alone and in combination with BMP-4, but again, cell death levels did not reach those found after RA and RA/BMP-4 treatment, indicating that besides the induction of RXRs, activation of RARs is equally essential for RA and RA/BMP-4-mediated apoptosis induction in WERI-Rb1 cells (Fig 5B). Thus, our data argue for a requirement of both RAR subtypes in RA and RA/BMP-4 apoptosis induction in WERI-Rb1 cells.

### RA and RA/BMP-4 apoptosis signaling in WERI-Rb1 cells involves RARα, RARß, RXRß and RXRγ

**Fig 6:**
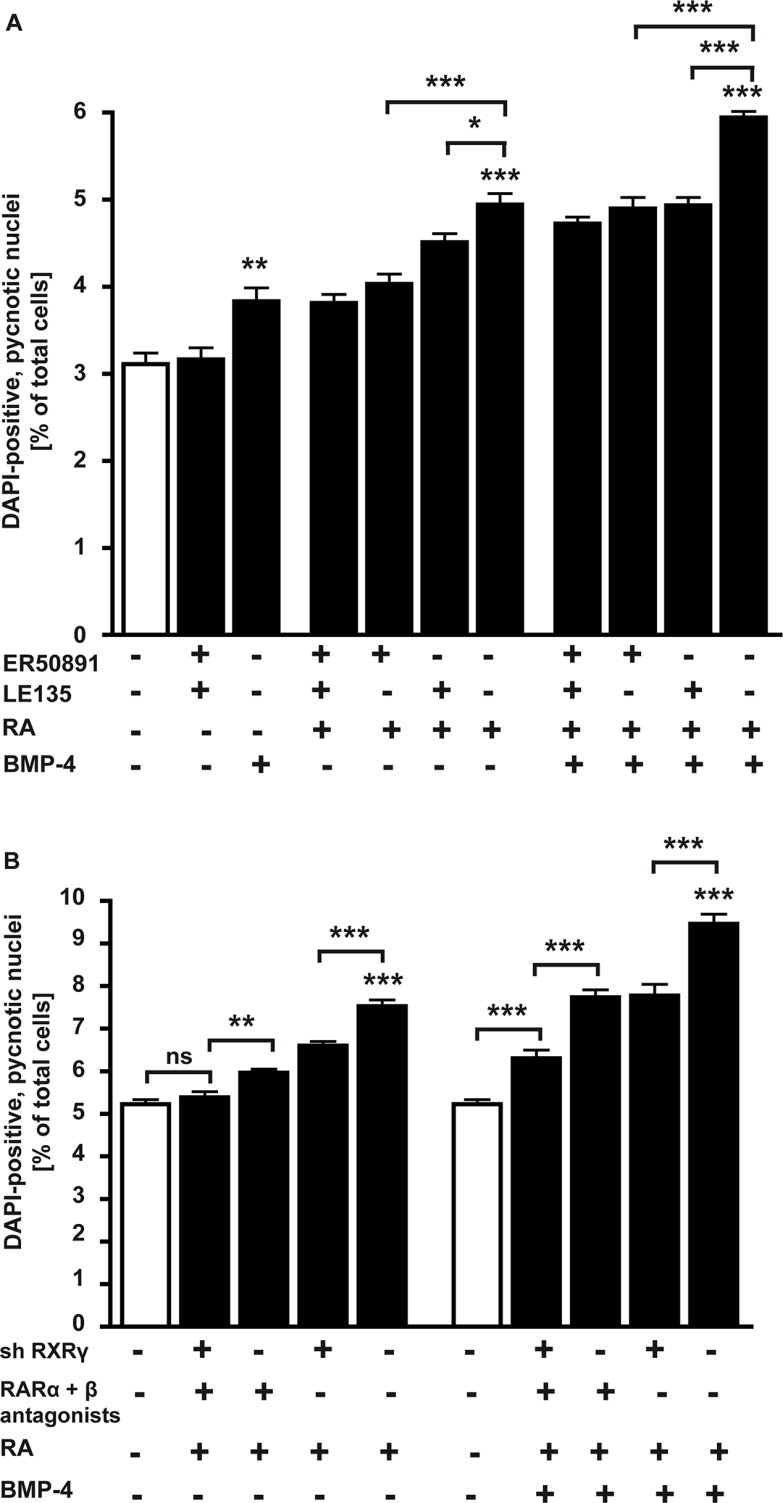
*RARα* and *RARß* antagonist (A) and *RXR*γ knockdown (B) studies. A, Application (+) of ER50891, a specific RARα antagonist or LE 135, a selectively RARß antagonist for 24 h disclosed the involvement of both RAR subtypes in RA/BMP-4 mediated apoptosis induction in WERI-Rb1 cells. B, WERI-Rb1 cells with a *RXR*γ knockdown (sh RXRγ) and non-silencing controls were treated for 24 h with RA or a combination of RA and BMP-4 in the presence (+) or absence (-) of a RARα antagonist (ER 50891) and a RARß antagonist (LE 135). Values are means from 3 independent assays ± SEM. *P < 0.05; **P < 0.01; ***P < 0.001 statistical differences calculated by one way Annova and Newman-Keuls Post test.

Each subtype of RARs has been implicated in the regulation of cancer development. Despite these studies, further investigation is needed to dissect the relevance of each receptor subtype in cancer cells in general and in retinoblastoma in particular. Compounds that possess specific binding properties, that is, receptor-selective retinoid antagonists, are useful tools for the precise elucidation of the mechanisms of RA actions. Using ER50891, a specific RARα antagonist [29] and LE 135, a selectively RARß antagonist [30], in the study presented, we could clearly demonstrate that apoptosis induction by RA/BMP-4 signaling in WERI-Rb1 cells requires both RAR subtypes, RARα and RARß (Fig 6A). Application of RA or RA/BMP-4 together with 10 μM ER50891 or 10 μM LE135 for 24 h significantly lowered the number of DAPI-positive, pycnotic nuclei compared to cells cultured in the absence of RAR antagonist. Higher concentrations (50 μM; S8 Fig) of ER50891 did not increase this effect (Fig A in S8 Fig); higher concentrations (50 μM) of LE135 likewise did not further reduce apoptosis levels (Fig B in S8 Fig). Combination of both RAR subtype antagonists and application together with RA or RA/BMP-4 did not result in a further decrease in apoptotic cell levels, suggesting that one or more additional RA receptors are involved.

It has been shown that RARs form heterodimers with RXRs, but our data indicated that *RXRα* is not inducible by RA or BMP-4 and *RXRß* seems to be exclusively regulated by BMP-4. Thus, RXRγ, additively induced by RA/BMP-4 treatment, was the most promising candidate to form heterodimers with RARα and RARß, shown to be involved in RA/BMP-4 apoptosis signaling.

As no subtype specific RXRß and RXRγ antagonists are commercially available, we designed an *RXRß* and an *RXRγ* knockdown (KD). Lentiviral transduction of WERI-Rb1 cells with sh *RXRß* and sh*RXRγ* lentiviral particles (S9 Fig) reduced the expression of *RXRß* by 78% (Fig A in S9 Fig) and *RXRγ* by 93% (Fig B in S9 Fig). Our functional study, in which we treated cells with RA or RA + BMP-4 in the presence or absence of both, RARα and RARß antagonists revealed that in WERI-Rb1 cells with a *RXRγ* KD apoptosis was less significantly induced compared to cells expressing the RXRγ gene (Fig 6B). Compared to untreated controls, RA was not able to induce apoptosis in WERI-Rb1-*RXRγ* KD cells treated with both RARα and RARß antagonists, strongly arguing for an important role of all three receptor subtypes in RA-mediated apoptosis induction. The combination of *RXRγ* KD with an application of RARα and RARß antagonists significantly decreased the number of apoptotic cells compared to antagonist treatment alone, but was not sufficient to completely abolish apoptosis induction by RA and BMP-4, suggesting that an additional receptor—RXRß and /or a BMP receptor—is required to mediate the additive apoptosis effects of a combined RA/BMP-4 treatment.

As *RXRß* expression is induced by BMP-4 (Fig B in S6 Fig), the additive effect in response to a combined RA/BMP-4 treatment might not only be dependent on RARα, RARß and RXRγ but also on RXRß. We thus designed an *RXRß* single and an *RXRß/RXRγ* double KD to address the question if the RXRß subtype receptor might also be crucial for a combined treatment induced apoptosis.

**Fig 7:**
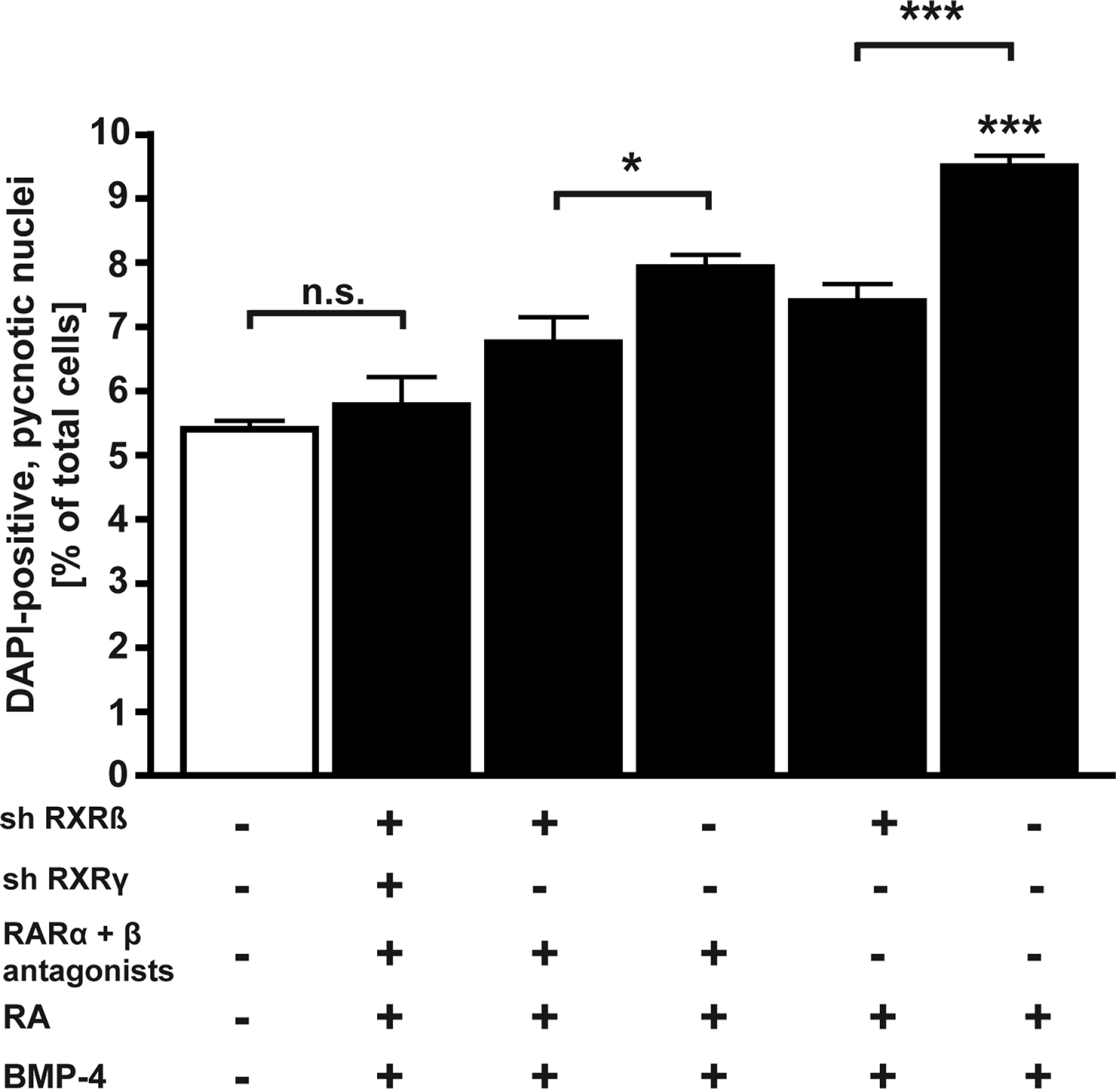
*RXRß* knockdown and *RXRß/RXRγ* double knockdown studies. WERI-Rb1 cells with an *RXR*ß knockdown (sh RXRß) and/or an *RXRγ* knockdown (sh RXRγ*)* and non-silencing controls were treated for 24 h with a combination of RA and BMP-4 in the presence (+) or absence (-) of a RARα antagonist (ER50891) and a RARß antagonist (LE135). Values are means ± SEM. *P < 0.05; ***P < 0.001 statistical differences calculated by one way Annova and Newman-Keuls Post test. n.s.: no significant statistical difference.

In WERI-Rb1 cells with an *RXRß* single KD, treatment with RA + BMP-4 induced significantly less apoptosis compared to control cells expressing the *RXRß* gene (Fig 7). In *RXRß/RXRγ* double KD cells, combined RA/BMP-4 treatment in the presence of RARα and RARß antagonists still slightly, but compared to control cells no longer significantly induces apoptosis, strongly arguing for the additional involvement of the RXRß receptor subtype in RA/BMP-4-mediated apoptosis induction.

## Discussion

Retinoids are already used in the treatment of epithelial cancer and promyelocytic leukemia [19; 39–42] and discussed for the therapy and / or chemoprevention of breast and gastric cancer [35; 43] as well as neuroblastoma [39]. The mechanisms of RA in cancer treatment include inhibition of cell proliferation, promotion of terminal tumor cell differentiation and induction of apoptosis [9]. It has been shown that RA has profound effects on cell growth and cause apoptosis in diverse tumor cells [1; 2; 44]. In the present study, RA clearly lowered the viability of WERI-Rb1 retinoblastoma cells and significantly increased the number of pycnotic nuclei, but had no detectable effect on the number of proliferating cells and no changes in the cell cycle profile could be detected. Our data are in full accordance with other studies likewise reporting that RA did not affect cell cycle distribution but triggered pronounced apoptosis in MCF-7 cells [45].

Former studies by our lab already demonstrated that BMP-4 induces apoptosis in WERI-Rb1 cells [20]. Though significant, BMP-4`s pro-apoptotic effect was low. In the present study, RA alone likewise only exerted small effects on apoptosis. Glozak and Rogers [21] likewise reported that RA alone induced apoptosis in only 10–15% of the cells and BMP-4 alone only minimally induced apoptosis, whereas the combination of RA and BMP-4 synergistically induced apoptosis in 40% of the P19EC cell population. Our results from experiments, in which we titrated RA against a fixed BMP-4 concentration and vice versa are in accordance with these findings, demonstrating that combined application of RA and BMP-4 results in increased cell death levels compared to single factor treatment. We likewise observed a significant, however, additive interaction of RA and BMP4 in the induction of apoptosis in different retinoblastoma cell lines. The reason for the distinct responses of RB and P19EC cells to RA, BMP-4 and combined treatment can be explained by the fact that cell lines with a completely different genetic, species and tissue-specific background were analyzed: on the one hand RB cells derived from primary human retinoblastomas with mutations or a complete loss of the *RB1* gene and on the other hand embryonic carcinoma cells derived from a murine teratocarcinoma.

Caspases, a family of cysteine proteases, are major executioners of apoptosis. Poulaki et al. [46] showed that the RB cells lines Y-79 and WERI-Rb1 do not express caspase-8. A former study by our group, however, demonstrated that BMP-4 mediated apoptosis in WERI-Rb1 cells is at least partially caspase-dependent [20]. It has been reported that RA- and RA/BMP-4 induced apoptosis in different tumor cells is related with an up-regulation and / or activation of caspase-3 [18; 22; 47]. In the study presented here, caspase blockage experiments and immunocytochmical stains revealed that RA and RA/BMP-4 induced apoptosis in WERI-Rb1 cells likewise involves cleavage and activation of caspase-3. In contrast to former findings [45], we did not detect an increase in full caspase-9 transcript levels upon administration of RA, BMP-4 or RA/BMP-4 double treatment (S10 Fig), however, observed a highly significant increase in caspase-9 cleavage activity following RA and BMP-4 administration, which was further increased by RA/BMP-4 double treatment. Our results are in accordance with former findings demonstrating that in P19 embryonal carcinoma cells, RA and BMP-4 induce the activation of caspase-9, being upstream of caspase-3 in the RA/BMP-4 mediated apoptosis enzyme cascade [22].

Apaf-1 is a cytoplasmic protein that forms one of the central hubs in the apoptosis regulatory network. Apaf-1 forms an oligomeric apoptosome that binds and cleaves caspase-9 [48]. Administration of RA had no effect on *Apaf1* mRNA expression in mammary carcinoma cells [45]. In our study, *Apaf-1* transcript levels likewise remained nearly unchanged upon application of RA, however, showed a significant up-regulation upon BMP-4 treatment. Co-application of RA in RA/BMP-4 double treatment approaches further increased *Apaf-1* mRNA levels, clearly indicating to an additive interaction of both factors in *Apaf-1* induction in WERI-Rb1 cells and indirectly confirming the involvement of caspases.

Previous studies found different receptor subtypes to be essential for apoptosis induction, depending on the carcinoma cell types. In P19 embryonal carcinoma cells RA/BMP-4 induced apoptosis is mediated through the activation of RARs and not RXRs [21]. Nagy et al. [38], by contrast, demonstrated that ligand activation of RXRs is essential for the induction of RA-mediated apoptosis in the leukemic myeloid precursor cell line HL-60 [38]. In the study presented, agonist studies revealed that both RARs and RXRs are involved in RA/BMP-4`s apoptosis induction in WERI-Rb1 retinoblastoma cells. Besides, we demonstrated that RA/BMP-4 apoptosis signalling in WERI-Rb1 cells requires the RA receptor subtypes RARα, RARß and RXRγ. Our results are in good accordance with data from Glozak and Rogers [21] showing that in P19 embryonal carcinoma cells RA/BMP-4 induced apoptosis is mediated through the specific activation of RARα [21]. The authors, however, found RARγ also to play a role [21], whereas in WERI-Rb1 cells *RARγ* is up-regulated by RA, but we could not augment this effect by co-application of BMP-4. The up-regulation of *RARß* is in good accordance with other studies showing that the level of *RARß* transcript increases dramatically in different cell types and tissues in response to RA [49; 50]. Using gene microarray chips, Li et al. [51] described the effects of RA on global gene expression patterns in WERI-Rb1 cells. The authors showed that treatment of WERI-Rb1 cells with ATRA for 48 h led to a significant up-regulation of RXRγ mRNAs. However, no changes in the expression level of the different RAR subtypes were detected by the microarray assay opposing data from our study, reporting on an up-regulation of RARα, RARβ and RARγ levels. The reason for these different findings is most likely the different duration of the ATRA treatment. Li et al. treated Weri-Rb-1 cells for 3h, 48 h and 7 days, while we chose a 24 h and 48 h treatment. In our setting the up-regulation of RARβ and RARγ mRNA levels by ATRA after 24 h is transient and no longer visible after 48 h. After 3h, the earliest time point of the study by Li et al. [51] the receptors are probably not yet induced and an up-regulation of RARα after 48 h not be high enough to induce a significant change in hybridization intensities in chip analyses.

In the present study, combined antagonist and knockdown approaches revealed that RA induced apoptosis is mediated through RARα, RARß, RXRß and RXRγ receptors. Though statistically not significant, combined application of RA/BMP-4 in *RXRß/RXRγ* double KD cells additionally treated with RARα and RARß antagonists, still slightly induces apoptosis. Thus, an additional receptor might be involved in mediating the additive effects of RA and BMP-4. As application of RA significantly up-regulated the mRNA level of BMPR II (S11 Fig), we hypothesize that this BMP receptor subtype might also contribute to RA/BMP-4 mediated cell death induction in WERI-Rb1 cells. Fujita et al. [22] likewise hypothesized that in P19 embryonic carcinoma cells up-regulation of BMPRII upon RA treatment makes the cells competent to respond to BMP-4 signals thus, inducing the synergistic effect of RA and BMP-4.

Summarizing, the present study demonstrates that a combined application of RA and BMP-4 amplifies the effect of single factor treatment and the signaling molecules involved, especially the receptors induced, might serve useful starting-points for future therapeutic approaches in retinoblastoma therapy, e.g. in combination with conventional chemotherapy. Retinoids are already used for the treatment of a number of diseases and new synthetic retinoids, specific for particular RA receptors may in the future be used for the treatment of other disorders including retinoblastoma. Ongoing studies will reveal further details on the signaling pathway underlying RA/BMP-4 mediated apoptosis induction in retinoblastoma cells and the effects of a combination with chemotherapeutic agents.

## Supporting Information

S1 Fig Effect of RA, BMP-4 and combined RA/BMP-4 treatment on cell proliferation in the retinoblastoma cell line WERI-Rb1 as determined by BrdU cell counts. n.s.: no significant statistical difference.
(TIF)

S2 Fig Apoptosis induction by RA, BMP-4 and combined treatment in WERI-Rb1 cells after long-time treatment. Cell counts of DAPI-positive, pycnotic nuclei were performed to determine apoptosis rates 48 h (Fig A,B) and 72 h (Fig C,D) after treatment with RA, BMP-4 or a combination of both, with (Fig B,D) and without (w/o; Fig A,C) restimulation after 24 h and 48 h. Forty-eight hours after application of RA, BMP-4 or a combination of both (Fig A,B), we detected higher apoptosis levels compared to those observed upon 24 h stimulation, but only in double treatment approaches and only after re-stimulation. Longer treatment (72h; Fig C,D) resulted in an increase in the number of apoptotic cells in single treatment approaches, whereas re-stimulation after 24 h and 48 h augmented the pro-apoptotic effect of combined factor treatment. **P < 0.01; ***P < 0.001 significant statistical differences compared to the control group calculated by one way Annova and Newman-Keuls Post test comparing all experimental groups.
(TIF)

S3 Fig Apoptosis induction by RA, BMP-4 and combined treatment in WERI-Rb1 cells as detected by Apo-BrdU TUNEL assay. 72 h after single stimulation with RA, BMP-4 or a combination of both, TUNEL-positive cells were counted manually and apoptosis rates were calculated as the percentage of total, Propidium iodide counterstained cells. **P < 0.01; ***P < 0.001 significant statistical differences calculated by one way Annova and Newman-Keuls Post test.
(TIF)

S4 Fig Apoptosis induction by RA, BMP-4 and combined treatment in Y-79 (Fig A), RB355 (Fig B), RBL-30 (Fig C) and RBL-15 (Fig D) cells. Cell counts of DAPI-positive, pycnotic nuclei were performed to determine apoptosis rates after treatment with RA, BMP-4 or a combination of both. 72 h treatment without restimulation resulted in a significant increase in the number of apoptotic cells in single as well as in double treatment approaches. *P < 0.05, **P < 0.01; ***P < 0.001 significant statistical differences calculated by one way Annova and Newman-Keuls Post test.
(TIF)

S5 Fig *RARα*, *ß*, *γ* and *RXRα*, *ß*, *γ* subtype expression in different RB cell lines. A healthy human retina pool served as a reference and was set as 1.
(TIF)

S6 Fig *RXRα* (Fig A) and *RXRß* (Fig B) transcript levels after RA, BMP-4 and RA/BMP-4 double treatment as revealed by RT-PCR. Cells treated with the solvents for RA and BMP-4 (see material and methods) served as controls (ctr.). *P < 0.05 statistical differences compared to the control group calculated by Student`s *t*-test.
(TIF)

S7 Fig Relative RAR subtypes transcript levels in WERI-Rb1 cells after RA, BMP-4 and RA/BMP-4 double treatment normalized against 18S rRNA (Fig A,C,E) and actin (Fig B,D,F). Compared to Real-time PCR analyses in which the housekeeping gene GAPDH was used as an internal control, the additive induction of RA receptor mRNA by RA/BMP-4 double treatment—exemplified for the induction *RARα*, *RARß* and *RARγ* after 24 h—persisted normalizing transcript levels against 18S rRNA or actin expression. Messenger RNA expression levels at the beginning of the treatment (0h) were used as a reference and set as 1.
(TIF)

S8 Fig *RARα* and *RARß* antagonist studies. Black bars: treatment with 10 μM ER50891 (*RARα* antagonist; Fig A) or LE135 (*RARß* antagonist; Fig B); grey bars: treatment with 50 μM of the respective antagonists. *P < 0.05; ***P < 0.001 statistical differences compared to the control group calculated by Student`s *t*-test. n.s.: no significant statistical difference.
(TIF)

S9 Fig *RXRß* and *RXRγ* transcript and protein levels after shRNA-mediated knockdown. Expression of *RXRß* and *RXRγ* mRNA and RXRγ protein levels after shRNA-mediated knockdown as revealed by Real-time-PCR (Fig A,B), RT-PCR (inset in Fig A) and Western Blot (Fig C).
(TIF)

S10 Fig Caspase-9 transcript levels upon administration of RA, BMP-4 and RA/BMP-4 double treatment. Cells treated with the solvents for RA and BMP-4 (see material and methods) served as controls (ctr.). n.s.: no significant statistical difference.
(TIF)

S11 Fig BMPR II expression levels upon RA, BMP-4 and RA/BMP-4 double treatment. Cells treated with the solvents for RA and BMP-4 (see material and methods) served as controls (ctr.). ***P < 0.001 statistical difference compared to the control group calculated by Student`s *t*-test. n.s.: no significant statistical difference.
(TIF)
