# Supplementary figures and images for: Additive Effects of Retinoic Acid (RA) and Bone Morphogenetic Protein 4 (BMP-4) Apoptosis Signaling in Retinoblastoma Cell Lines
Source: PLoS One. 2015 Jul 14;10(7):e0131467. doi: 10.1371/journal.pone.0131467 (PMC4501735; doi:10.1371/journal.pone.0131467)

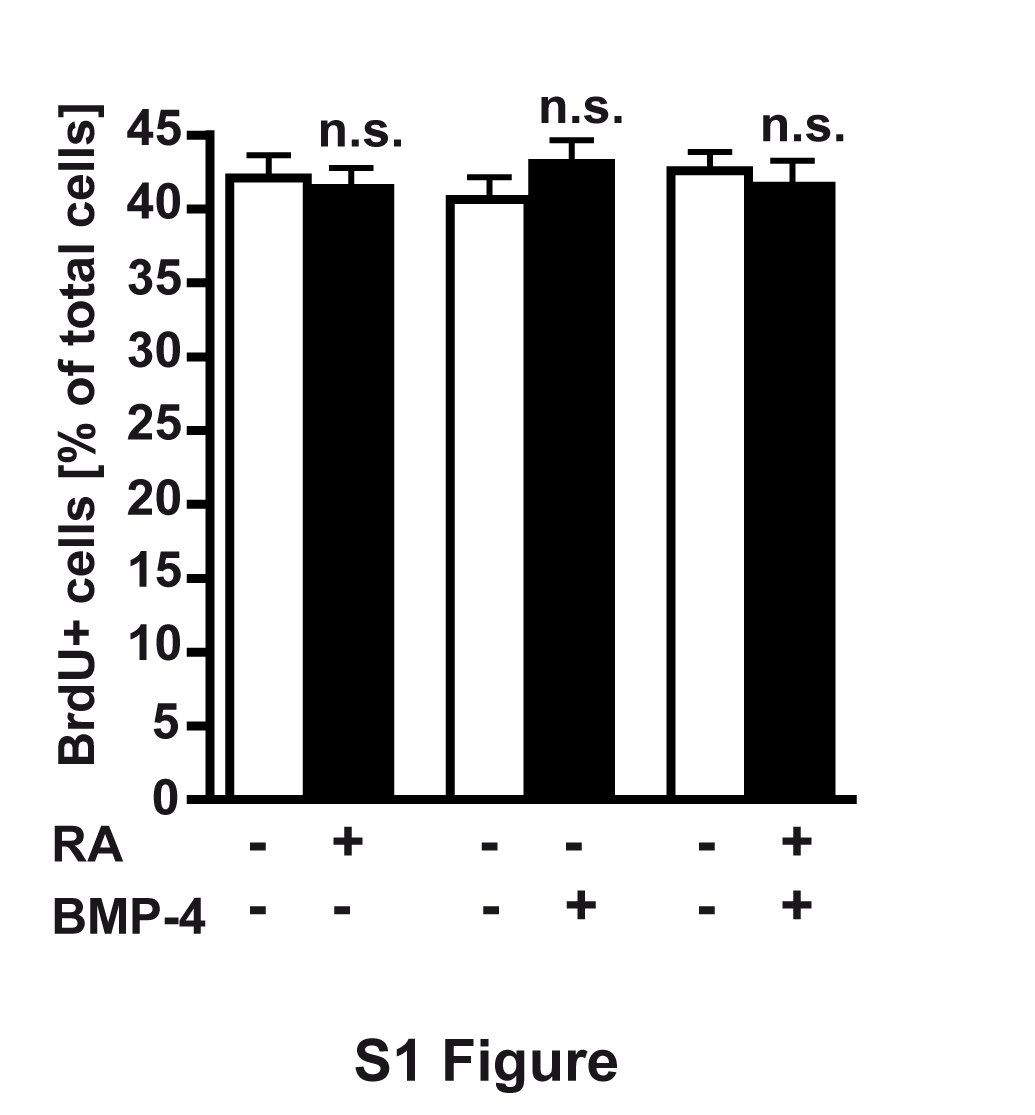

Supplement: S1 Fig — n.s.: no significant statistical difference. (TIF) [file pone.0131467.s001.tif]

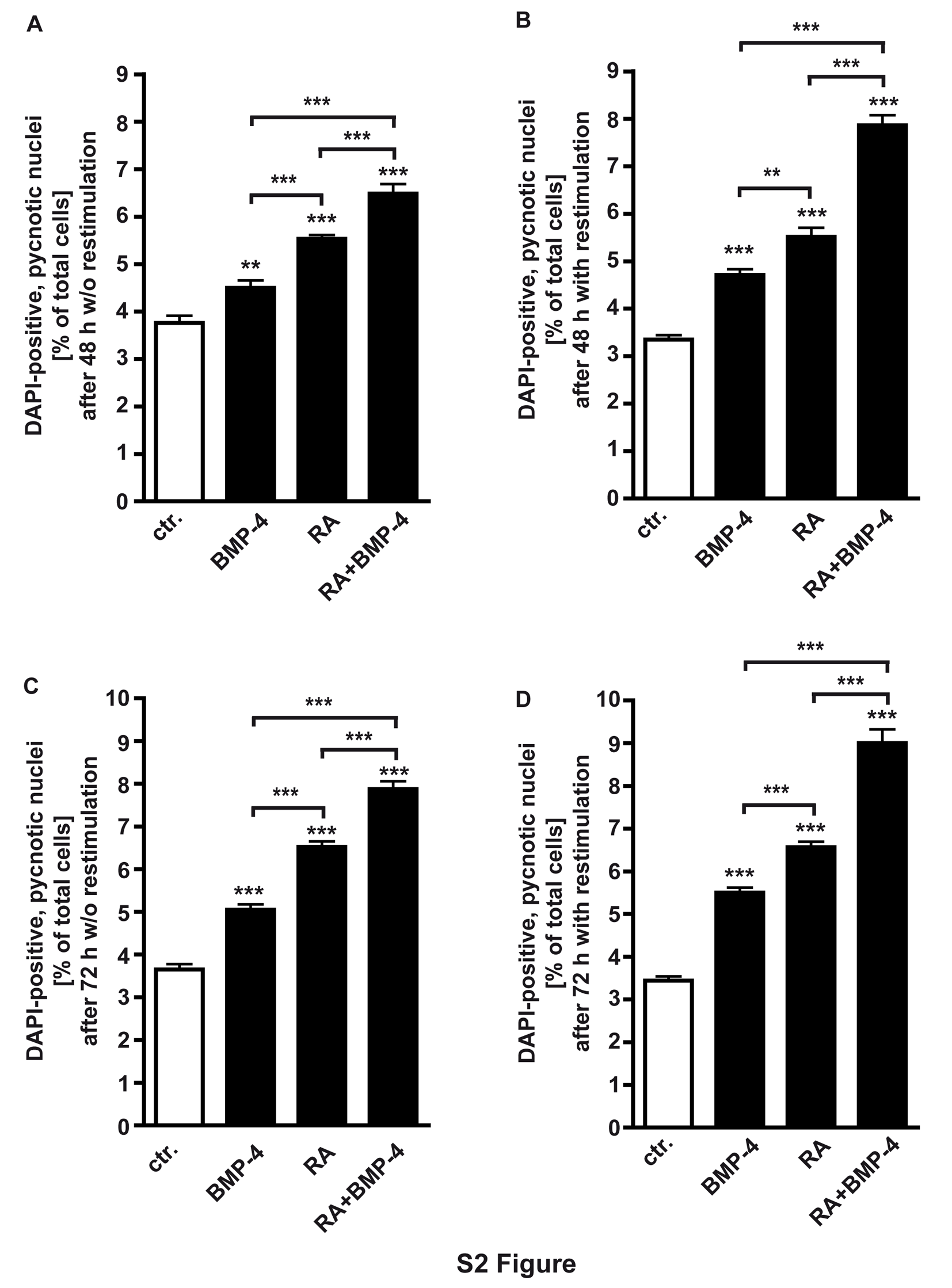

Supplement: S2 Fig — Cell counts of DAPI-positive, pycnotic nuclei were performed to determine apoptosis rates 48 h (Fig A,B) and 72 h (Fig C,D) after treatment with RA, BMP-4 or a combination of both, with (Fig B,D) and without (w/o; Fig A,C) restimulation after 24 h and 48 h. Forty-eight hours after application of RA, BMP-4 or a combination of both (Fig A,B), we detected higher apoptosis levels compared to those observed upon 24 h stimulation, but only in double treatment approaches and only after re-stimulation. Longer treatment (72h; Fig C,D) resulted in an increase in the number of apoptotic cells in single treatment approaches, whereas re-stimulation after 24 h and 48 h augmented the pro-apoptotic effect of combined factor treatment. **P < 0.01; ***P < 0.001 significant statistical differences compared to the control group calculated by one way Annova and Newman-Keuls Post test comparing all experimental groups. (TIF) [file pone.0131467.s002.tif]

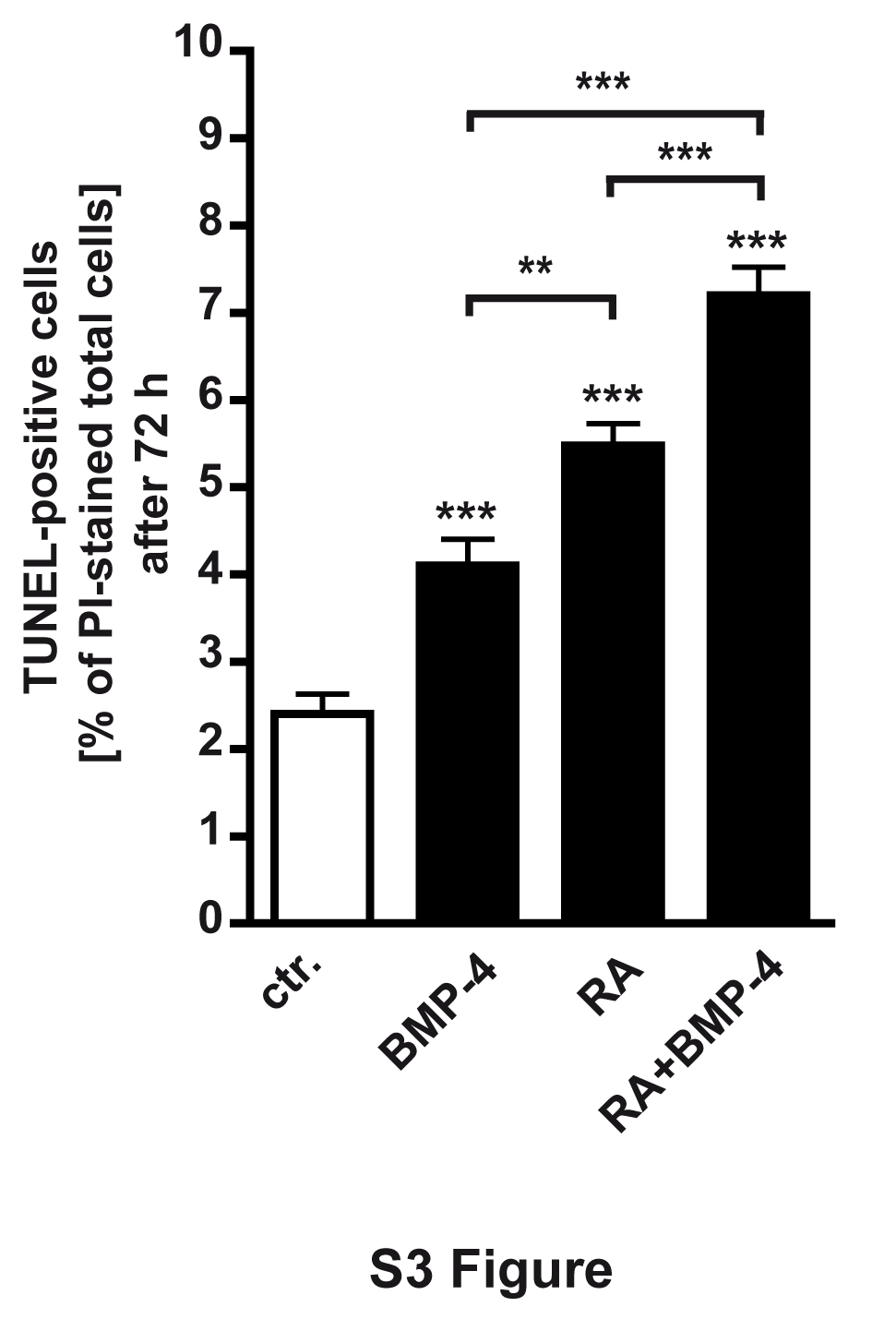

Supplement: S3 Fig — 72 h after single stimulation with RA, BMP-4 or a combination of both, TUNEL-positive cells were counted manually and apoptosis rates were calculated as the percentage of total, Propidium iodide counterstained cells. **P < 0.01; ***P < 0.001 significant statistical differences calculated by one way Annova and Newman-Keuls Post test. (TIF) [file pone.0131467.s003.tif]

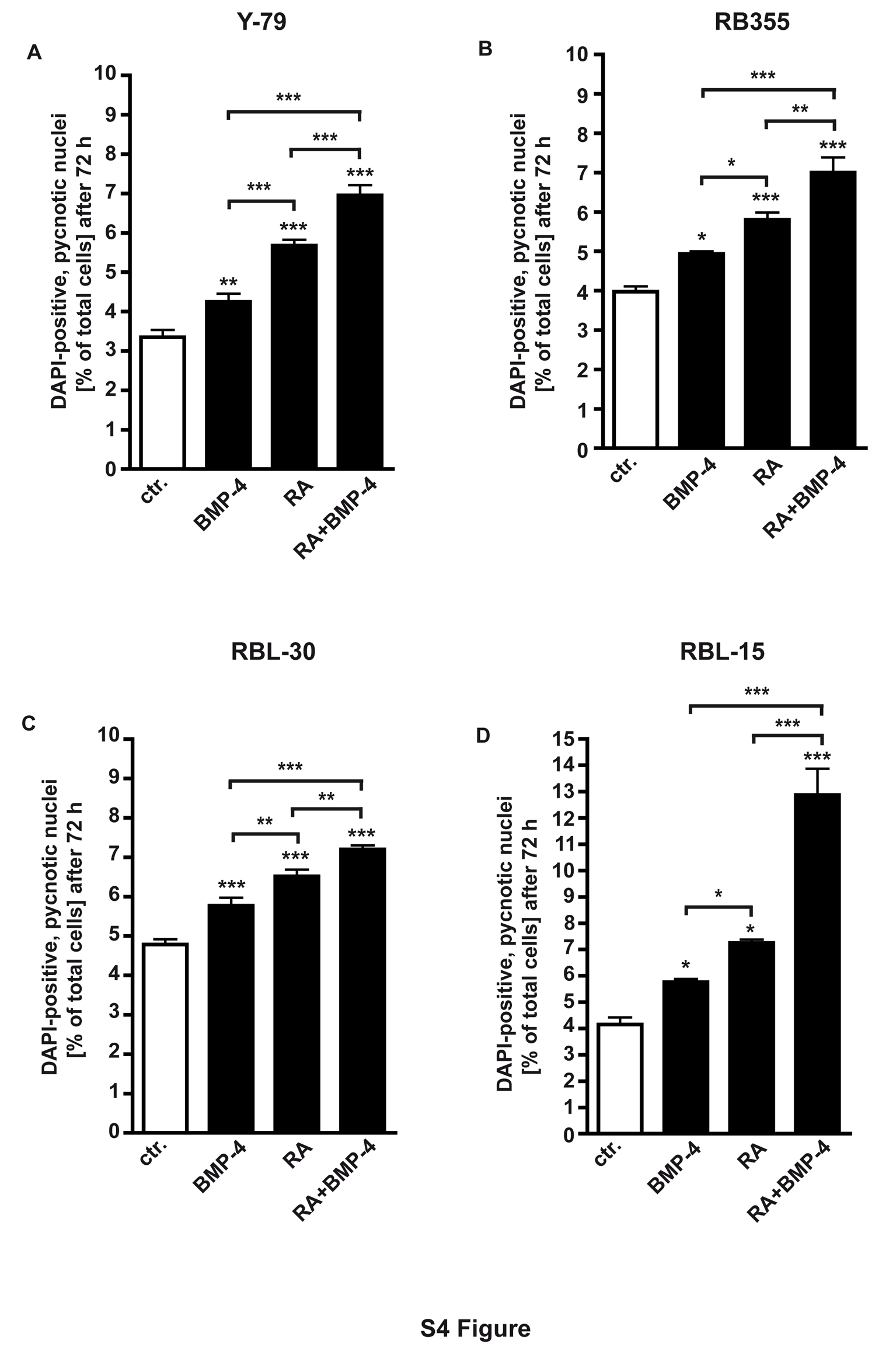

Supplement: S4 Fig — Cell counts of DAPI-positive, pycnotic nuclei were performed to determine apoptosis rates after treatment with RA, BMP-4 or a combination of both. 72 h treatment without restimulation resulted in a significant increase in the number of apoptotic cells in single as well as in double treatment approaches. *P < 0.05, **P < 0.01; ***P < 0.001 significant statistical differences calculated by one way Annova and Newman-Keuls Post test. (TIF) [file pone.0131467.s004.tif]

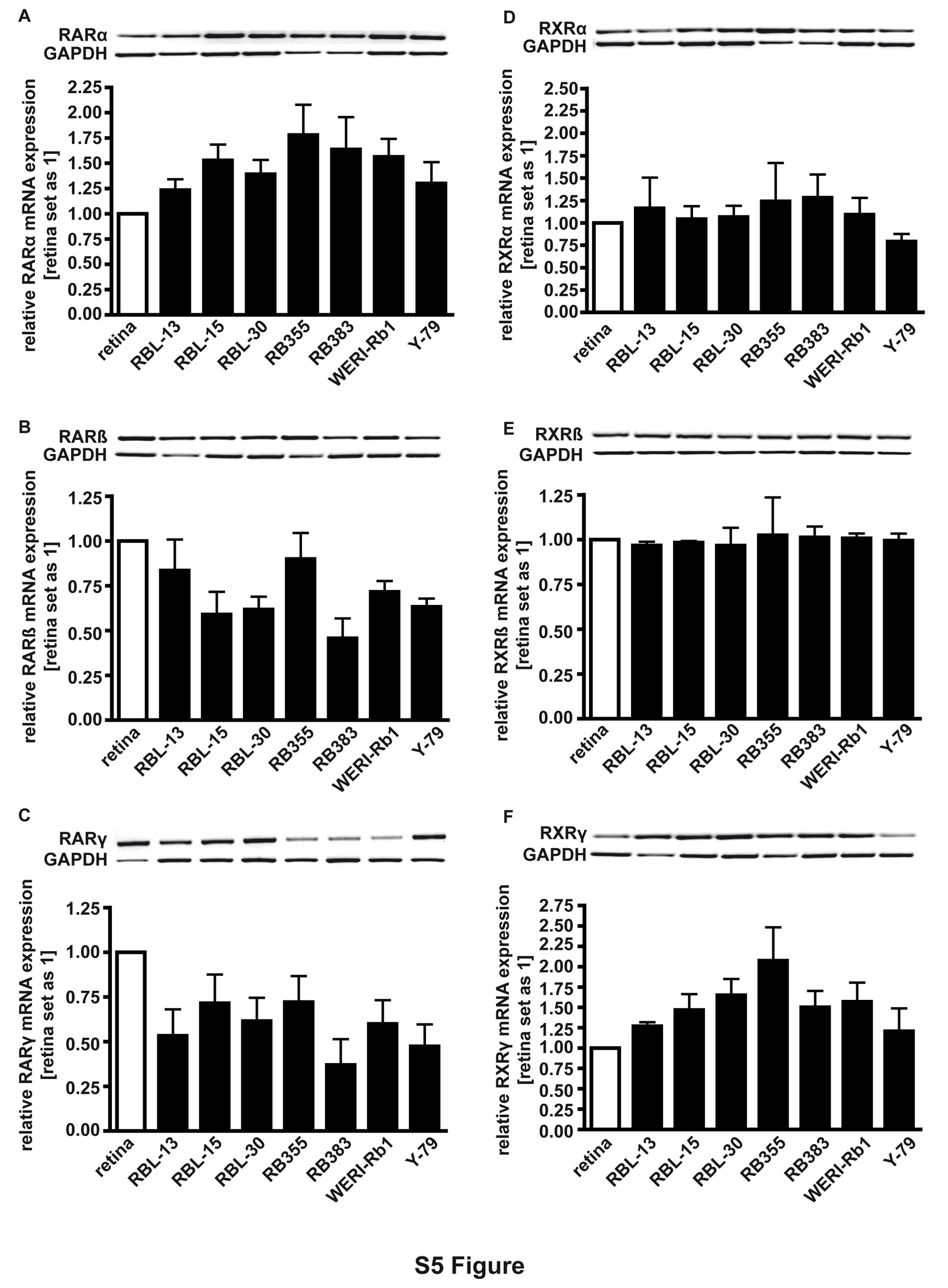

Supplement: S5 Fig — A healthy human retina pool served as a reference and was set as 1. (TIF) [file pone.0131467.s005.tif]

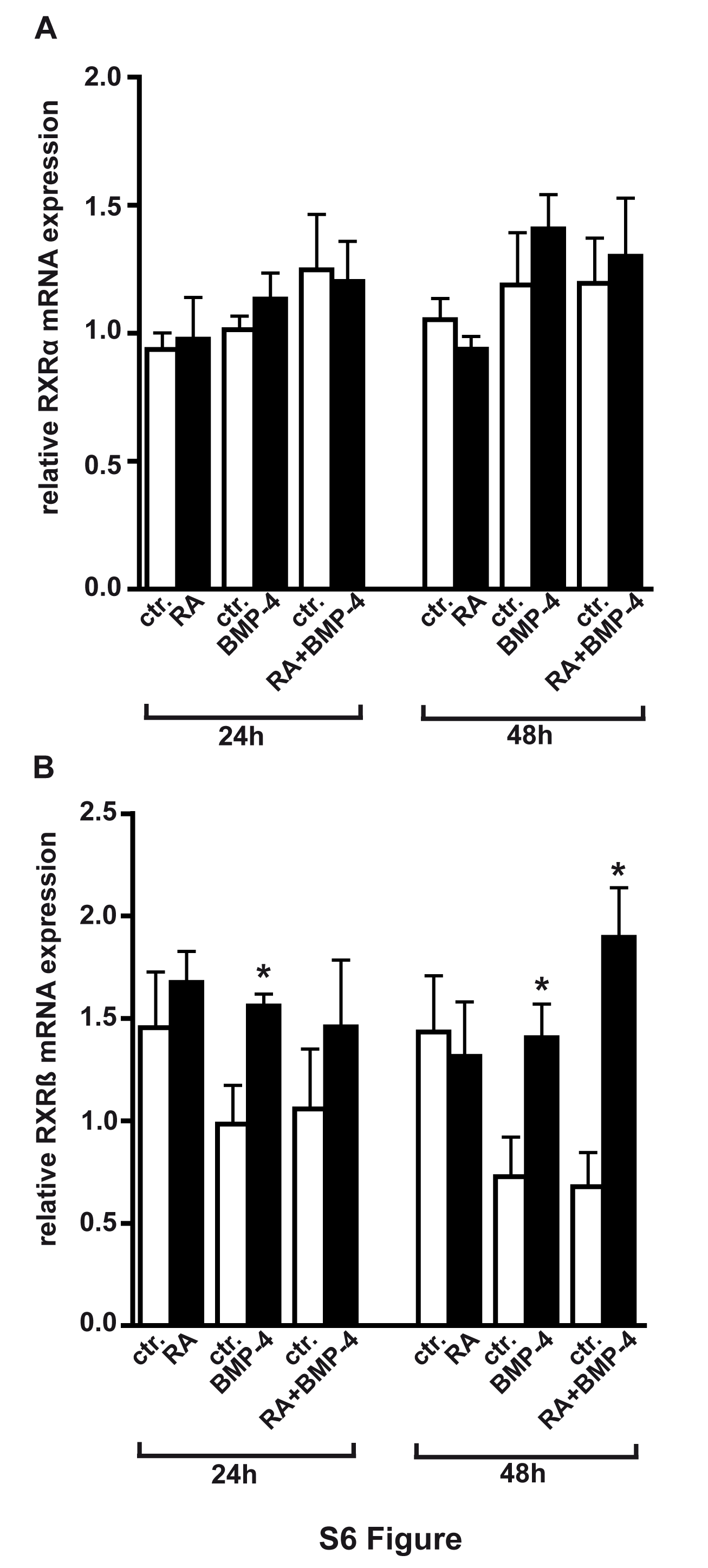

Supplement: S6 Fig — Cells treated with the solvents for RA and BMP-4 (see material and methods) served as controls (ctr.). *P < 0.05 statistical differences compared to the control group calculated by Student`s t-test. (TIF) [file pone.0131467.s006.tif]

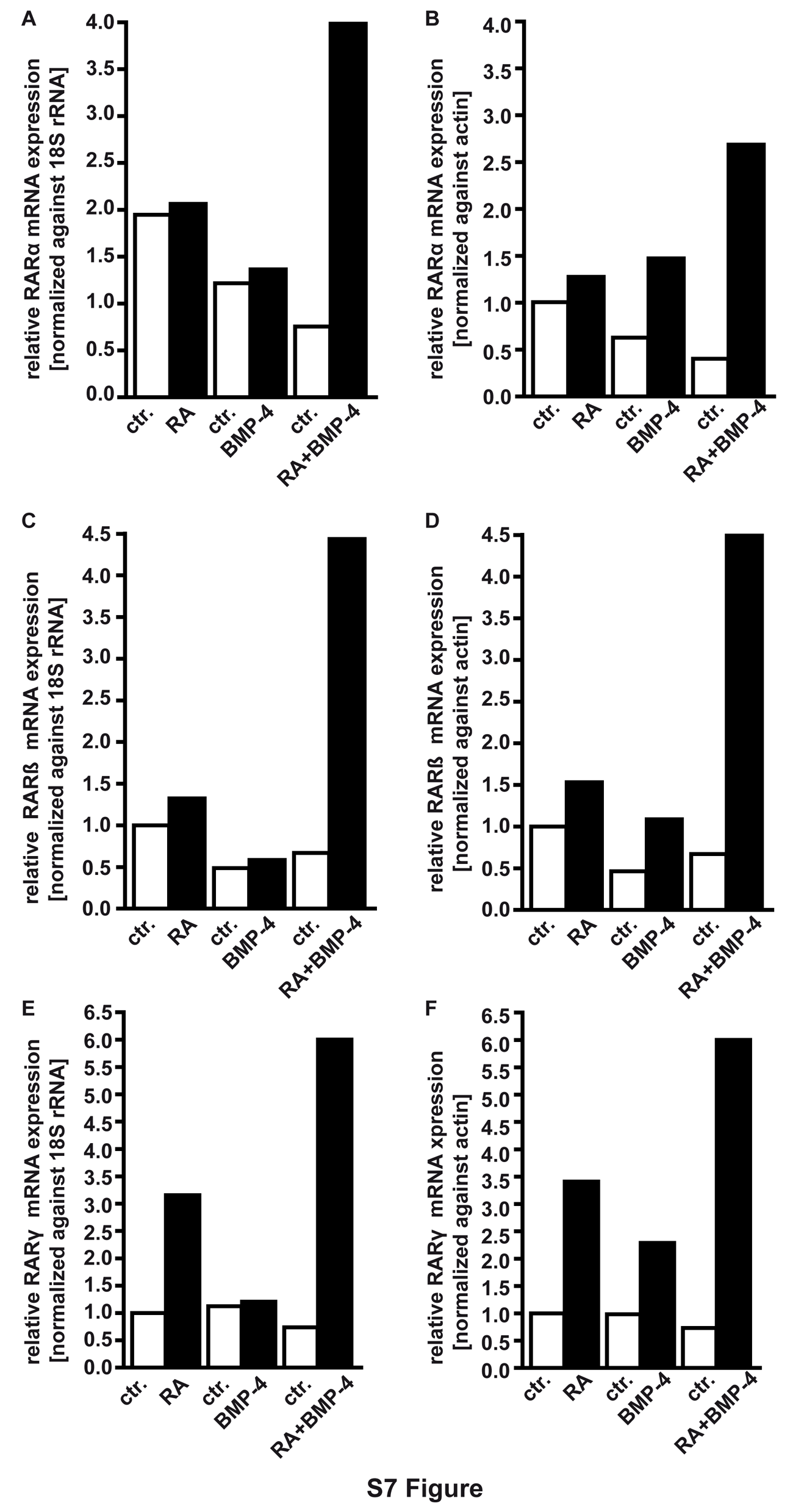

Supplement: S7 Fig — Compared to Real-time PCR analyses in which the housekeeping gene GAPDH was used as an internal control, the additive induction of RA receptor mRNA by RA/BMP-4 double treatment—exemplified for the induction RARα, RARß and RARγ after 24 h—persisted normalizing transcript levels against 18S rRNA or actin expression. Messenger RNA expression levels at the beginning of the treatment (0h) were used as a reference and set as 1. (TIF) [file pone.0131467.s007.tif]

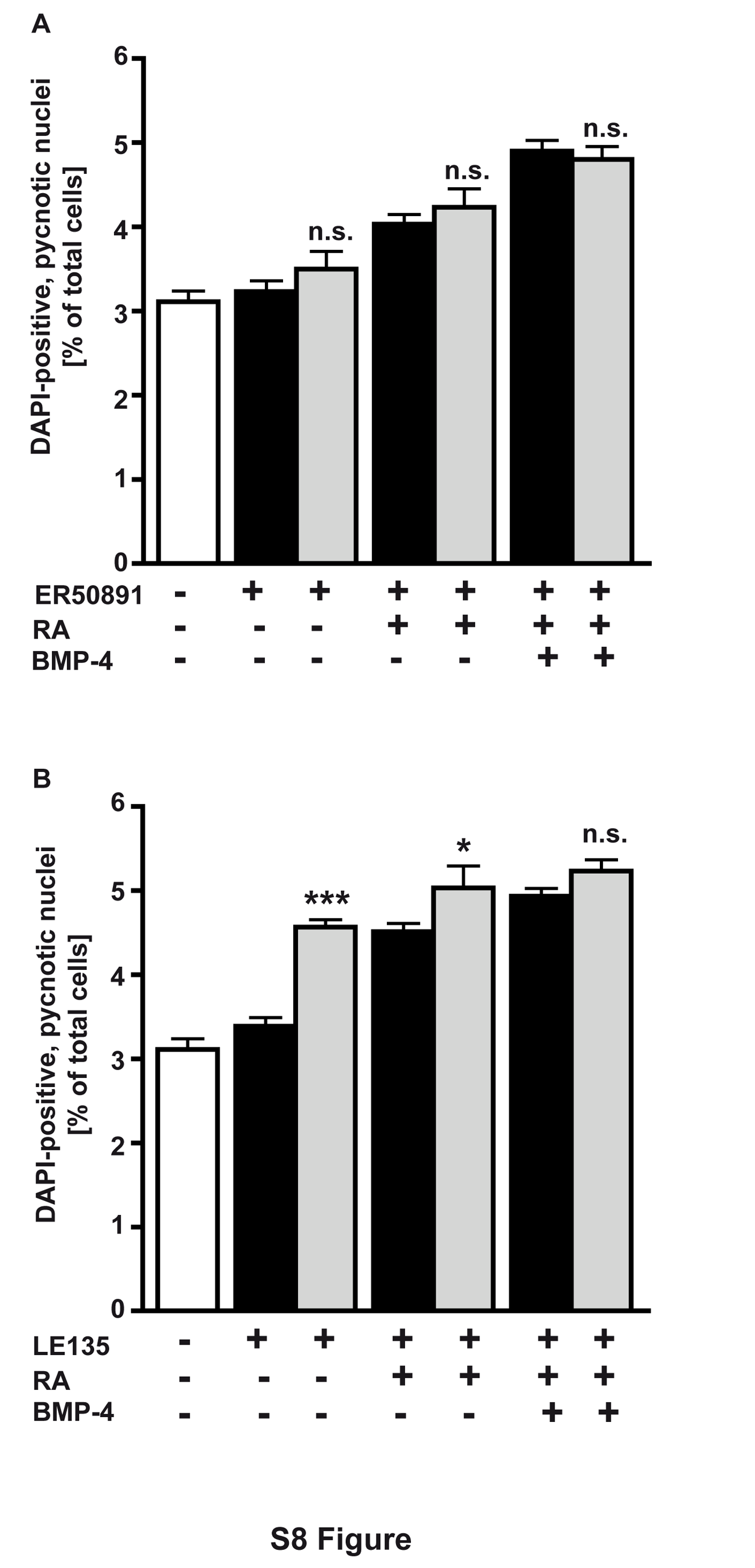

Supplement: S8 Fig — Black bars: treatment with 10 μM ER50891 (RARα antagonist; Fig A) or LE135 (RARß antagonist; Fig B); grey bars: treatment with 50 μM of the respective antagonists. *P < 0.05; ***P < 0.001 statistical differences compared to the control group calculated by Student`s t-test. n.s.: no significant statistical difference. (TIF) [file pone.0131467.s008.tif]

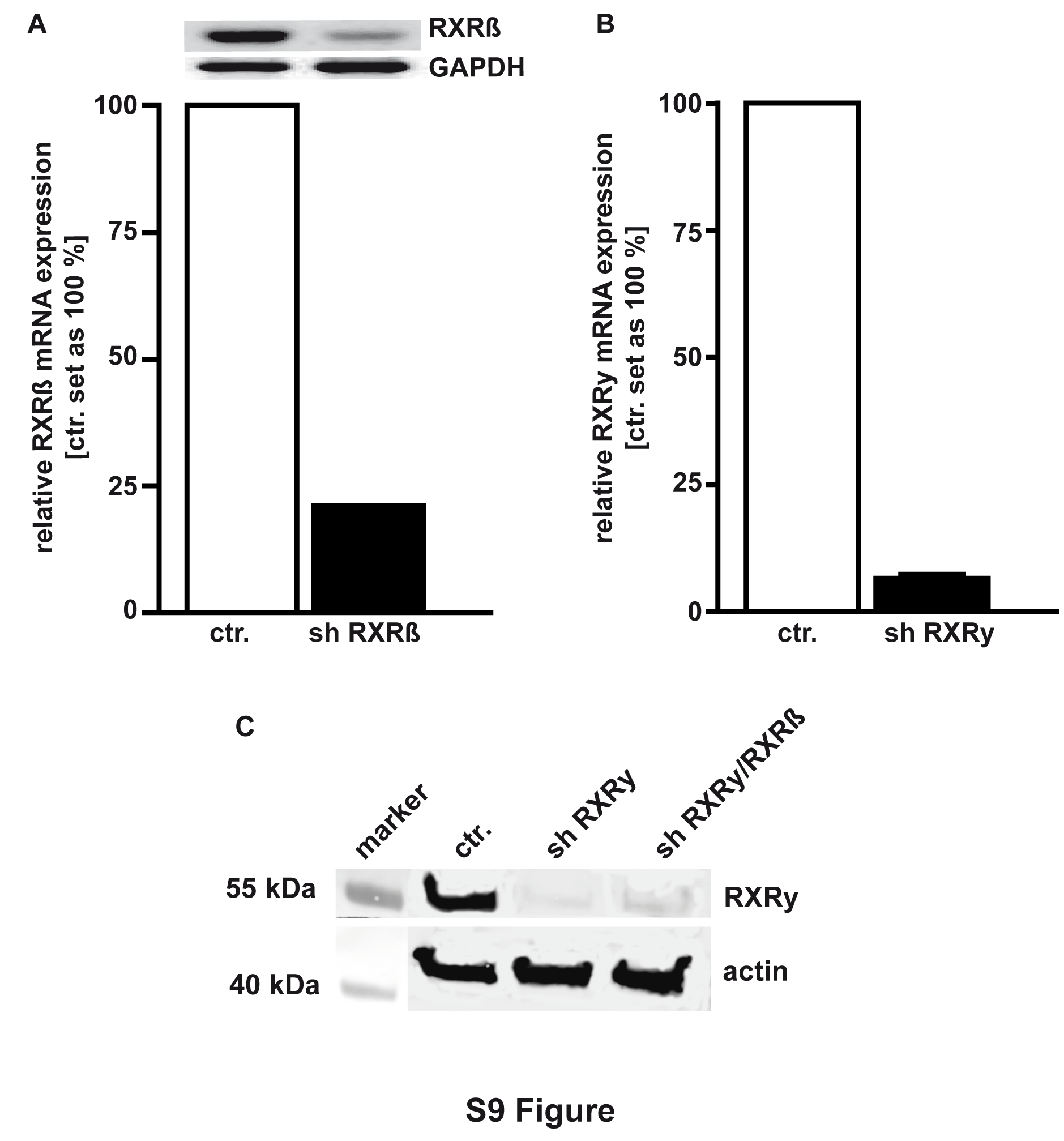

Supplement: S9 Fig — Expression of RXRß and RXRγ mRNA and RXRγ protein levels after shRNA-mediated knockdown as revealed by Real-time-PCR (Fig A,B), RT-PCR (inset in Fig A) and Western Blot (Fig C). (TIF) [file pone.0131467.s009.tif]

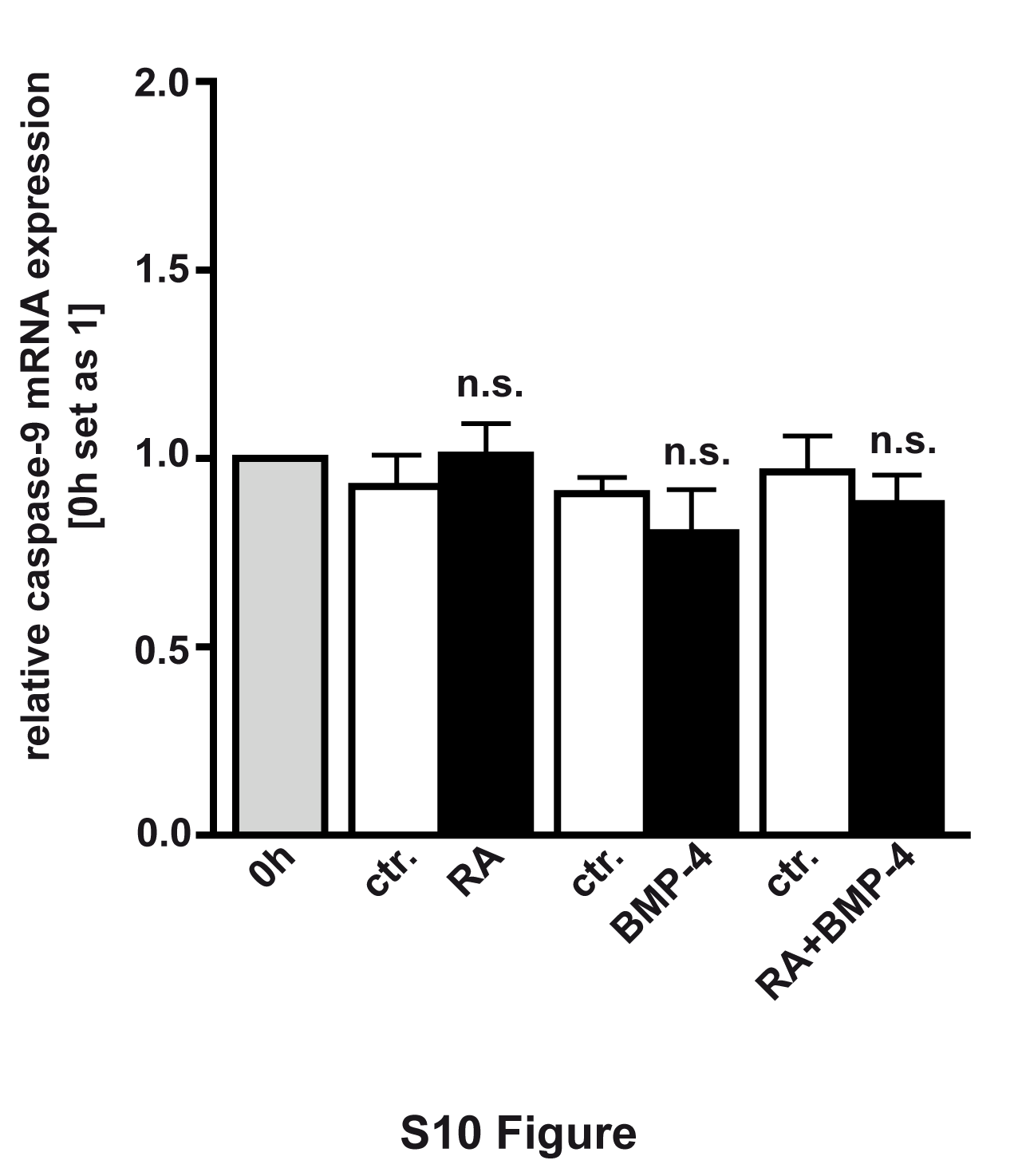

Supplement: S10 Fig — Cells treated with the solvents for RA and BMP-4 (see material and methods) served as controls (ctr.). n.s.: no significant statistical difference. (TIF) [file pone.0131467.s010.tif]

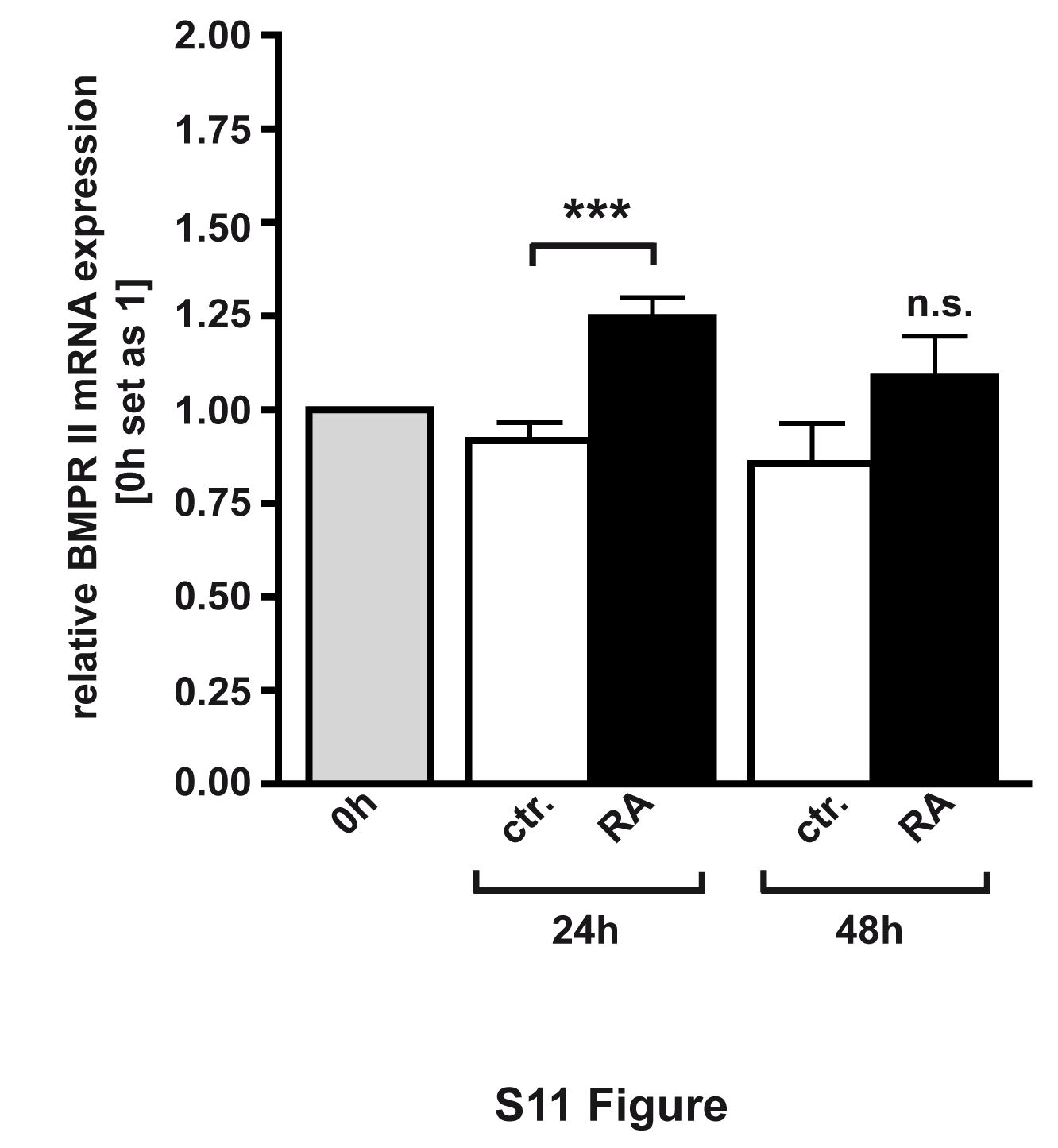

Supplement: S11 Fig — Cells treated with the solvents for RA and BMP-4 (see material and methods) served as controls (ctr.). ***P < 0.001 statistical difference compared to the control group calculated by Student`s t-test. n.s.: no significant statistical difference. (TIF) [file pone.0131467.s011.tif]
